# Tumor Invasive Border Index (TIBI) in colorectal cancer: linking infiltrative morphology to molecular insights

**DOI:** 10.1002/path.70087

**Published:** 2026-06-18

**Authors:** Akseli Kehusmaa, Jouni Härkönen, Huayi Li, Päivi Sirniö, Ville K Äijälä, Henna Karjalainen, Meeri Kastinen, Vilja V Tapiainen, Tuomo Mantere, Vesa‐Matti Pohjanen, Hanna Elomaa, Onni Sirkiä, Nicolas Pasquier, Maarit Ahtiainen, Olli Helminen, Erkki‐Ville Wirta, Taneli T Mattila, Outi Lindgren, Janette Savela, Jukka Rintala, Sanna Meriläinen, Juha Saarnio, Tero Rautio, Toni T Seppälä, Jan Böhm, Jukka‐Pekka Mecklin, Markus J Mäkinen, Anne Tuomisto, Johanna Ivaska, Juha P Väyrynen

**Affiliations:** ^1^ Translational Medicine Research Unit, Medical Research Center Oulu Oulu University Hospital, and University of Oulu Oulu Finland; ^2^ Department of Pathology, Hospital Nova of Central Finland Well Being Services County of Central Finland Jyväskylä Finland; ^3^ Faculty of Health Sciences, A.I. Virtanen Institute for Molecular Sciences University of Eastern Finland Kuopio Finland; ^4^ Turku Bioscience Centre University of Turku and Åbo Akademi University Turku Finland; ^5^ Research Program in Systems Oncology University of Helsinki Helsinki Finland; ^6^ Department of Environmental and Biological Sciences University of Eastern Finland Kuopio Finland; ^7^ Central Finland Biobank Well Being Services County of Central Finland Jyväskylä Finland; ^8^ Department of Gastroenterology and Alimentary Tract Surgery Tampere University Hospital Tampere Finland; ^9^ Faculty of Medicine and Health Technology, Tampere University and Tays Cancer Centre Tampere University Hospital Tampere Finland; ^10^ Department of Gastrointestinal Surgery, Helsinki University Central Hospital University of Helsinki Helsinki Finland; ^11^ Applied Tumor Genomics, Research Program Unit University of Helsinki Helsinki Finland; ^12^ Department of Education and Research Wellbeing Services County of Central Finland Jyväskylä Finland; ^13^ Faculty of Sport and Health Sciences University of Jyväskylä Jyväskylä Finland; ^14^ Department of Life Technologies University of Turku Turku Finland; ^15^ InFLAMES Research Flagship University of Turku Turku Finland; ^16^ Western Finnish Cancer Center (FICAN West) University of Turku Turku Finland; ^17^ Foundation for the Finnish Cancer Institute Helsinki Finland

**Keywords:** tumor border configuration, colorectal cancer, tumor biomarkers, tumor invasion, epithelial‐mesenchymal transition

## Abstract

Tumor border configuration influences colorectal cancer (CRC) prognosis, yet its molecular determinants remain unclear and existing assessment criteria have faced challenges with reproducibility. We introduce the Tumor Invasive Border Index (TIBI), a novel and reproducible method that quantifies the proportion of tumor stroma and adipose tissue within a hotspot at the deepest point of invasion. TIBI was evaluated in two CRC cohorts (*n* = 1,100 and *n* = 776) and analyzed in relation to tumor and patient features. Molecular correlates of an infiltrative growth pattern were explored in The Cancer Genome Atlas (TCGA) CRC cohorts (*n* = 350), with key features validated independently. High TIBI, indicating an infiltrative border, was associated with advanced disease, tumor budding, lymphovascular invasion, and an immune microenvironment characterized by lower M1‐like macrophage and granulocyte densities. High TIBI independently predicted higher CRC‐specific mortality, with multivariable hazard ratios of 1.52 (95% CI 1.07–2.17) in cohort 1 and 2.45 (95% CI 1.37–4.37) in cohort 2. Molecular analysis revealed associations with mismatch repair proficiency, *TP53* and *KRAS* mutations, MYC signaling downregulation, and epithelial‐mesenchymal transition upregulation. *L1CAM* and *DSG3* were among the genes showing high expression in infiltrative tumors. As experimental validation, we identified a CRC cell line with high expression of *L1CAM* and *DSG3* and demonstrated that silencing them reduced invasion *in vitro*. A TIBI‐associated gene signature also predicted infiltrative growth and adverse outcome in the TCGA gastric cancer cohort. These findings highlight molecular characteristics of tumor border configuration and establish TIBI as a clinically relevant tumor biomarker. © 2026 The Author(s). *The Journal of Pathology* published by John Wiley & Sons Ltd on behalf of The Pathological Society of Great Britain and Ireland.

## Introduction

Colorectal cancer (CRC) is the third most common cancer worldwide (1.9 million new cases in 2022) [1], with a 5‐year survival rate of approximately 65% in high‐income countries, strongly dependent on stage [2, 3]. Because staging alone incompletely predicts outcome, additional molecular and histological features are used to guide adjuvant treatment, including *KRAS*/*BRAF* mutations, mismatch repair (MMR) status, tumor budding, perineural invasion, and lymphovascular invasion [4, 5, 6, 7, 8, 9, 10].

Tumor border configuration (expanding versus infiltrating) has long been associated with prognosis but poor reproducibility has limited clinical uptake. In studies using Jass criteria, the proportion of tumors classified as ‘infiltrating’ has ranged widely (17–70%) across observers [11], despite consistent associations with adverse tumor characteristics and worse survival [10, 12, 13, 14, 15]. Infiltrative border has been linked with advanced stage, increased tumor budding, and reduced immune infiltration at the invasive border [11, 16], yet the biological processes shaping the tumor border morphology remain incompletely defined [17, 18, 19].

We developed a quantitative, reproducible metric for tumor border assessment, termed the Tumor Invasive Border Index (TIBI). It estimates the proportion of tumor stroma and adipose tissue within a defined hotspot at the point of deepest invasion. We evaluated inter‐rater agreement for TIBI versus Jass criteria, applied TIBI to two large CRC cohorts (*n* = 1,100 and 776), explored molecular correlates in The Cancer Genome Atlas (TCGA), and validated key features in our own cohorts. Finally, we tested the impact of silencing two TIBI‐associated genes (*L1CAM* and *DSG3*) on CRC cell invasion *in vitro*.

## Materials and methods

### Ethical approval and patient consent

For cohort 1, the study was conducted under approval from the Regional Medical Research Ethics Committee of the Wellbeing Services County of North Ostrobothnia (25/2002, 42/2005, 122/2009, 37/2020), Biobank Borealis (BB‐2017_1012), and Fimea (FIMEA/2022/001941). For cohort 2, the study was conducted under approval from the Regional Medical Research Ethics Committee of the Wellbeing Services County of Central Finland (Dnro 13U/2011, 1/2016, 8/2020, 2/2023), Central Finland Biobank (BB23‐0172), and Fimea (Dnro FIMEA/2023/001573, 4/2023). In cohort 1, all participants gave written informed consent for the study. For cohort 2, the need to obtain informed consent from the study patients was waived (Dnro FIMEA/2023/001573, 4/2023). The study was conducted in accordance with the Declaration of Helsinki (https://www.wma.net/policies-post/wma-declaration-of-helsinki/, last accessed 14 May 2026).

### Study population

The study included two cohorts of patients with CRC undergoing surgical resection at either Central Finland Central Hospital in Jyväskylä (cohort 1, *n* = 1,343; years 2000–2015) or Oulu University Hospital (cohort 2, *n* = 1,011; years 2006–2020) [20, 21, 22]. Cohort 1 was collected retrospectively and cohort 2 prospectively. Patients receiving preoperative radiotherapy or chemotherapy were excluded (cohort 1: *n* = 243; cohort 2: *n* = 235). Additionally, patients who died within 30 days of the surgery (*n* = 37 for cohort 1, *n* = 5 for cohort 2) were excluded from survival analysis.

Median follow‐up for censored cases was 9.5 years (IQR 6.8–13.4) in cohort 1 and 5.9 years (IQR 3.8–8.8) in cohort 2. Survival analysis was limited to the first 10 years post‐surgery. The primary endpoint was cancer‐specific survival (CSS, time from surgery to CRC‐specific death); the secondary endpoint was overall survival (OS, time from surgery to any death). Clinical data were collected from medical records, and follow‐up data from clinical records and Statistics Finland (https://stat.fi/en, last accessed 14 May 2026).

For molecular correlates, TCGA colon and rectal adenocarcinomas (COAD/READ) were analyzed. Of these, 350 cases with representative formalin‐fixed paraffin‐embedded or frozen H&E sections showing a visible tumor border were included (supplementary material, File S1). The TIBI‐associated molecular signature was additionally evaluated in TCGA gastric adenocarcinoma (stomach adenocarcinoma [STAD]) cases (*n =* 415). Patient selection is summarized in the supplementary material, Figure S1.

### Histopathological analysis

TNM stage was defined during routine diagnostics using the Union for International Cancer Control (UICC)/American Joint Committee on Cancer (AJCC) criteria [23]. Digitized H&E slides were utilized in additional analyses. Tumor grade followed WHO 2019 (low versus high) [24]. Lymphovascular invasion was defined as tumor cells within vascular spaces. Tumor budding was scored as low/intermediate/high following the International Tumor Budding Consensus Conference (ITBCC) criteria [25].

### Assessment of tumor border configuration

Tumor border configuration was assessed on digitized H&E‐stained slides. To establish criteria for the TIBI, we assessed the relative abundance of various tissue categories within a 4 mm diameter hotspot (5× field) positioned at the deepest point of tumor invasion. For cases with multiple slides, the slide with deepest invasion was chosen. Within the hotspot, TIBI was calculated as the ratio of total area of fibrous stroma and adipose tissue to the total tissue surface area, which included fibrous stroma, adipose tissue, tumor epithelium, and mucus. Other tissue categories, such as blood vessels, necrosis, muscle, nerves, and whitespace, were excluded. Detailed instructions are provided in the supplementary material, File S2. All assessments were performed by observers masked to outcome data.

To assess reproducibility and refine TIBI criteria, 10 observers (five pathologists and five researchers with expertise in CRC histopathology) scored TIBI in 30 samples. Prior to the analysis, observers received a brief introduction to the method and the TIBI evaluation manual. For comparison, observers also classified tumor border configuration using the Jass criteria as either expanding (tumor border relatively well circumscribed with no to little dissection of surrounding tissue) or infiltrating (no distinctive tumor border with widespread dissection of surrounding tissue) [26].

Following the initial inter‐rater agreement study, we revised the TIBI manual to address the cases with the greatest discrepancies and incorporated observer feedback to enhance clarity. A second inter‐rater agreement analysis was then conducted using three raters (two gastrointestinal pathologists and one researcher) on 30 different cases. In this round, TIBI was compared with tumor budding (ITBCC criteria).

### Immunohistochemistry and immune cell analyses

Tissue microarrays (TMAs) contained four 1.0 mm cores/patient, two tumor center, two invasive margin [20]. *BRAF* V600E mutation status and MMR status were immunohistochemically assessed as previously described [20]. For this study, additional immunohistochemistry was performed with Leica Bond RX/3 platforms (Leica Biosystems, Nussloch, Germany) using the BOND Polymer Refine Detection kit (Leica DS9800), BOND Epitope Retrieval Solution 2 (Leica AR9640, 30 min, 100°C) pretreatment, and antibodies specific for TP53 (DO‐7, 1:200, Epredia, Portsmouth, NH, USA), L1CAM (14.10, 1:50, BioLegend, San Diego, CA, USA), DSG3 (EPR14101, 1:80, Abcam, Cambridge, UK), MYC (EP121, 1:30, Cell Marque, Rocklin, CA, USA), and KRT17 (E3, 1:20, Leica) [27]. Immunohistochemistry targets were selected to validate, at the protein level, prioritized TIBI‐associated genes and pathway markers identified in our transcriptomic/genetic analyses. Markers were selected based on consistency of association with TIBI, relevance to invasive biology, and availability of validated antibodies for immunohistochemistry. Successful analysis was achieved for 1,099 (TP53) cases in cohort 1 and 760 (TP53/L1CAM/MYC) or 759 (DSG3/KRT17) cases in cohort 2.

Immune profiling was based on three multiplex immunohistochemistry assays (cyclic staining with 3‐Amino‐9‐ethylcarbazole chromogen) combined with digital image analyses in cohort 1 (supplementary material, Figure S2) [28, 29, 30]. Quantified cell populations included CD3^+^ T cells, CD20^+^CD79A^+^ B cells, CD20^−^CD79A^+^ plasma cells, M1‐like and M2‐like macrophages, CD14^+^HLA‐DR^+^ mature monocytic cells, CD14^+^HLA‐DR^−^ immature monocytic cells, CD66B^+^ granulocytes, and tryptase^+^ mast cells. A four‐marker (M1: CD86, HLA‐DR; M2: CD163, CD206) polarization index was used for macrophage phenotyping [28]. Assays were successful in 1,045 (myeloid), 1,065 (T cells/macrophages), and 1,070 (B cells/plasma cells) cases.

### Single‐cell RNA sequencing analysis

We analyzed the CRC single‐cell RNA sequencing (scRNA‐seq) dataset GSE178341 (https://www.ncbi.nlm.nih.gov/geo/query/acc.cgi?acc=GSE178341, last accessed 14 May 2026) with cell‐type annotations from the associated publication [31]. Data were processed using scCustomize (v.2.1.2, https://cran.r-project.org/web/packages/scCustomize/index.html, last accessed 14 May 2026), Seurat (v.1.3.8) [32] and Harmony (v.1.2.0) [33], including cells with detected features within the range of 200–2,500, while excluding cells with high mitochondrial read content (> 10%). A total of 62 available tumor samples were analyzed. The data were normalized and scaled with the Seurat *NormalizeData* and *ScaleData* functions. Clustering was conducted using the Louvain algorithm, and data were visualized using uniform manifold approximation and projection (UMAP).

### Bulk mRNA and DNA sequencing analysis

TCGA COAD/READ clinical, gene expression, copy‐number, and mutation data were obtained from the GDC PanCanAtlas resource; MSI Sensor scores from cBioPortal (https://www.cbioportal.org/, last accessed 14 May 2026); and Cancer Cell Line Encyclopedia data for somatic mutations and gene expression from DepMap (https://depmap.org/portal/, last accessed 1 May 2025). Tumor mutational burden (TMB) was calculated as somatic mutations per 1,000,000 bases, assuming a 30,000,000 base pair exome. Consensus Molecular Subtypes (CMS) were determined using R package CMScaller [34].

In TCGA‐COAD and TCGA‐READ, ‘high’ and ‘low’ TIBI categories were used in evaluating differences between infiltrative and expanding tumors. Differential expression analysis was performed using limma and gene set enrichment analysis (GSEA) for Hallmark pathways was conducted using fGSEA [35]. Copy‐number analysis was conducted with GISTIC2 [36].

A transcriptomic TIBI score (TIBI‐t) was derived from genes significantly different between TIBI categories (*p* < 0.01). To enhance cross‐tumor applicability we only retained genes with highest expression in stromal cells in an independent scRNA‐seq dataset, originally generated from a separate cohort of 62 patients (supplementary material, Figure S3). Weights were assigned based on log2 fold changes, and TIBI‐t was the mean of (log2 expression × weight).

For validation, TIBI was visually assessed on TCGA‐STAD tumors in the bottom/top 4% of TIBI‐t (supplementary material, File S3), and TIBI‐t distributions were compared across TIBI categories. High TIBI‐t was defined as greater than the upper quartile.

### Optical genome mapping

We profiled structural variation in 35 tumor samples using the Saphyr system and DLE‐1 labeling chemistry (Bionano Genomics, San Diego, CA, USA), following the manufacturer's protocol [27]. Data were processed with Rare Variant Pipeline (Bionano Solve v3.8) and visualized in Bionano Access (v1.8.1). Default confidence thresholds and size filters were applied. The analysis focused on rare structural variants (absent from the Bionano Genomics control database) and copy‐number variants overlapping/near the 13q12.2 locus (to follow up GISTIC results).

### Cell culture

SW837 cells (RRID:CVCL_1729) (American Type Culture Collection, ATCC, Manassas, VA, USA) were cultured in RPMI‐1640 with 10% fetal bovine serum (FBS). Transfections were performed using Opti‐MEM (Thermo Fisher Scientific, Waltham, MA, USA, 31985–047) and Lipofectamine RNAiMAX (Thermo Fisher Scientific, 56532) following the manufacturer protocols. For *L1CAM* knockdown we used *L1CAM* Human siRNA Oligo Duplex (SR422542, Origene) siL1CAM‐1 (SR302635B; 5′‐GGAAUGUAAAAUACACCGUGACUU‐3’) and siL1CAM‐2 (SR302635C; 5′‐CGGAUACAAUGUGACGUACUGGAGG‐3′). For *DSG3* knockdown, we used a siRNA pool with three specific oligos (sc‐43115, Santa Cruz Biotechnology, Dallas, TX, USA). For invasion assay, 5 × 10^4^ cells were seeded onto Matrigel‐coated Transwell inserts (354480, Corning, Corning, NY, USA); after 24 h, invaded cells were fixed with 4% paraformaldehyde (PFA) (10 min) and stained with 0.5% crystal violet (15 min). The dye was eluted with 10% acetic acid and absorbance was measured at 595 nm using a microplate reader (Multiskan Ascent, Thermo Scientific). Cell proliferation was assessed using CellTiter‐Glo Luminescent Cell Viability Assay (Promega, Madison, WI, USA) in 96‐well plates (4 × 10^3^ cells/well; *n* = 8 replicates) at indicated time points.

### Statistical analyses

Statistical analyses were performed using IBM SPSS Statistics for Windows (IBM Corp., Armonk, NY, USA, v. 29.0) and R statistical programming (R Foundation for Statistical Computing, Vienna, Austria, version 4.3.1). Statistical significance was defined as a two‐sided *p* < 0.05.

Receiver operating characteristics (ROC) analysis was used to evaluate area under the curve (AUC) for different tissue types at the invasive margin contributing to prognostic significance for CSS and to explore potential combinations to define TIBI. TIBI was defined as a continuous area fraction (0–100%) within the annotated hotspot. For categorical analyses, TIBI was additionally grouped into three categories (< 40%, 40–67%, and > 67%). These cutoffs were chosen to create approximately equal‐sized groups in cohort 1 and were rounded to simple thresholds to facilitate reproducible visual scoring. These same cutoffs were then applied unchanged to cohort 2.

Inter‐rater agreement for TIBI was evaluated as a continuous variable using Spearman correlation coefficient. For categorical analysis, agreement was evaluated using the kappa statistic (*κ*) for three‐category (< 40%, 40–67%, and > 67%) and two‐category (≤ 67%, > 67%) TIBI, as well as the Jass criteria.

Associations between TIBI categories and tumor and patient characteristics were evaluated using the *χ* ^2^ test, and the Kruskal‐Wallis test was used for immune cell densities. Analyses involving immune cell densities were additionally stratified by MMR status, considering its strong influence on the tumor immune microenvironment [37].

Kaplan–Meier curves with log‐rank test were used to assess differences in survival across TIBI categories and by L1CAM and DSG3 expression. For these analyses, tumors were grouped into the three TIBI categories described above and dichotomized as negative or positive for L1CAM and DSG3. To compare the prognostic potential of TIBI with tumor budding, univariable and multivariable Cox proportional hazards regression models were utilized to calculate hazard ratios (HRs) for TIBI and tumor budding categories. Because TIBI and tumor budding both reflect invasive‐front morphology, we assessed their inter‐relationship. Spearman correlations between TIBI and tumor budding were moderate (continuous: *ρ* = 0.44 and 0.39 in cohorts 1 and 2; three‐tier categorization: *ρ* = 0.39 and 0.35, respectively). We therefore present Cox models both without and with adjustment for tumor budding to evaluate incremental prognostic information.

## Results

### TIBI is a reproducible method for assessing tumor border configuration

To establish criteria for TIBI, we quantified tissue components (tumor epithelium, fibrous stroma, smooth muscle, adipose tissue, normal mucosa, extracellular mucin, necrosis, peripheral nerve, blood vessel, neutrophilic abscess, tertiary lymphoid tissue, whitespace) within a 4 mm diameter hotspot at the deepest invasive margin of 1,100 tumors in cohort 1. ROC analysis identified tumor epithelium, fibrous stroma, and adipose tissue as the tissue types most predictive of CSS (supplementary material, Figure S4), and TIBI was defined as the hotspot area fraction of (fibrous stroma + adipose tissue) divided by (tumor epithelium + fibrous stroma + adipose tissue + extracellular mucin).

Reproducibility was tested in two rounds. First, 10 observers independently scored 30 cases (supplementary material, Figure S5). Continuous TIBI showed substantial agreement (mean Spearman *ρ* = 0.70). When TIBI was categorized into three groups (low: < 40%, intermediate: 40–67%, high: > 67%), the mean κ was 0.46, while for the binary classification (low to intermediate: ≤ 67%, high: > 67%) mean *κ* was 0.67. Jass expanding/infiltrating classification was less reproducible (*κ* = 0.34). Cases with discrepancies were then reviewed and observer feedback was incorporated into an updated TIBI evaluation manual.

In a second inter‐rater agreement study, three observers scored 30 new cases, comparing TIBI with tumor budding (ITBCC criteria). Agreement for TIBI exceeded that for tumor budding: continuous *ρ* = 0.88 versus 0.72; three‐tier *κ* = 0.69 versus 0.51; binary *κ* = 0.80 versus 0.56. These findings indicate that tumor border configuration can be reproducibly evaluated on H&E sections using the TIBI criteria.

### High TIBI is associated with advanced disease stage, lymphovascular invasion, tumor budding, and MMR proficient status

Using the newly established criteria, TIBI was evaluated for two large cohorts of patients with stage I−IV CRC (Table 1). High TIBI was associated with high T, N, and M stages, lymphovascular invasion, MMR proficient status, and high tumor budding (both cohorts: *p* < 0.001). High TIBI was inversely associated with *BRAF* V600E mutation (cohort 1: *p* < 0.001; cohort 2: *p* = 0.007).

**Table 1:** Demographic and clinical characteristics of colorectal cancer cases according to tumor infiltration, as classified by Tumor Invasive Border Index (TIBI).

	Cohort 1					Cohort 2				
		TIBI					TIBI			
Characteristic	Total *n*	Low (< 40%)	Intermediate (40%–67%)	High (> 67%)	*p* value	Total *n*	Low (< 40%)	Intermediate (40%–67%)	High (> 67%)	*p* value
All cases	1,100 (100%)	341 (31%)	387 (35%)	372 (34%)		776 (100%)	220 (28%)	302 (39%)	254 (33%)	
Sex					0.68					0.67
Male	557 (51%)	174 (31%)	199 (36%)	184 (33%)		412 (53%)	116 (28%)	166 (40%)	130 (32%)	
Female	543 (49%)	167 (31%)	188 (35%)	188 (35%)		364 (47%)	104 (29%)	136 (37%)	124 (34%)	
Age (years)					0.048					0.15
< 65	290 (26%)	87 (30%)	91 (31%)	112 (39%)		233 (30%)	61 (26%)	81 (35%)	91 (39%)	
65–75	381 (35%)	108 (28%)	154 (40%)	119 (31%)		285 (37%)	85 (30%)	119 (42%)	82 (29%)	
> 75	429 (39%)	146 (34%)	142 (33%)	141 (33%)		258 (33%)	74 (29%)	102 (40%)	82 (32%)	
Tumor location					0.072					0.014
Proximal colon	536 (49%)	184 (34%)	175 (33%)	177 (33%)		323 (42%)	99 (31%)	128 (40%)	96 (30%)	
Distal colon	404 (37%)	113 (28%)	144 (36%)	147 (37%)		205 (26%)	67 (33%)	64 (31%)	74 (36%)	
Rectum	160 (15%)	44 (28%)	68 (43%)	48 (30%)		248 (32%)	54 (22%)	110 (44%)	84 (34%)	
AJCC T class					< 0.001					< 0.001
T1	58 (5.3%)	24 (41%)	30 (52%)	4 (6.9%)		49 (6%)	20 (41%)	25 (51%)	4 (8%)	
T2	167 (15%)	68 (41%)	76 (46%)	23 (14%)		180 (23%)	74 (41%)	88 (49%)	18 (10%)	
T3	679 (62%)	209 (31%)	235 (35%)	235 (35%)		435 (56%)	98 (23%)	158 (36%)	179 (41%)	
T4	196 (18%)	40 (20%)	46 (23%)	110 (56%)		112 (14%)	28 (25%)	31 (28%)	53 (47%)	
AJCC N class					< 0.001					< 0.001
N0	626 (57%)	255 (41%)	230 (37%)	141 (23%)		455 (59%)	170 (37%)	196 (43%)	89 (20%)	
N1	275 (25%)	49 (18%)	110 (40%)	116 (42%)		192 (25%)	33 (17%)	74 (39%)	85 (44%)	
N2	199 (18%)	37 (19%)	47 (24%)	115 (58%)		129 (17%)	17 (13%)	32 (25%)	80 (62%)	
AJCC M class					< 0.001					< 0.001
M0	947 (86%)	318 (36%)	344 (36%)	285 (30%)		691 (89%)	207 (30%)	283 (41%)	201 (29%)	
M1	153 (14%)	23 (15%)	43 (28%)	87 (57%)		85 (11%)	13 (15%)	19 (22%)	53 (62%)	
Tumor grade					0.031					0.096
Low grade	903 (82%)	264 (29%)	330 (37%)	309 (34%)		665 (86%)	188 (25%)	268 (40%)	209 (31%)	
High grade	197 (18%)	77 (39%)	57 (29%)	63 (32%)		111 (14%)	32 (29%)	34 (31%)	45 (41%)	
Lymphovascular invasion					< 0.001					< 0.001
No	858 (78%)	319 (37%)	321 (37%)	218 (25%)		429 (55%)	165 (38%)	184 (43%)	80 (19%)	
Yes	242 (22%)	22 (9.1%)	66 (27%)	154 (64%)		347 (45%)	55 (16%)	118 (34%)	174 (50%)	
Tumor budding ITBCC					< 0.001					< 0.001
Low	827 (75%)	315 (38%)	301 (36%)	211 (26%)		541 (70%)	193 (36%)	227 (42%)	121 (22%)	
Intermediate	156 (14%)	20 (13%)	57 (37%)	79 (51%)		129 (17%)	19 (15%)	41 (32%)	69 (53%)	
High	117 (11%)	6 (5.1%)	29 (25%)	82 (70%)		106 (14%)	8 (8%)	34 (32%)	64 (60%)	
MMR status					< 0.001					< 0.001
MMR proficient	931 (85%)	256 (28%)	329 (35%)	346 (37%)		652 (84%)	163 (25%)	257 (39%)	232 (34%)	
MMR deficient	169 (15%)	85 (50%)	58 (34%)	26 (15%)		124 (16%)	57 (46%)	45 (36%)	22 (18%)	
*BRAF* status					< 0.001					0.007
Wild‐type	916 (83%)	266 (29%)	326 (36%)	324 (35%)		662 (85%)	174 (26%)	262 (40%)	226 (34%)	
Mutant	182 (17%)	75 (41%)	59 (32%)	48 (26%)		107 (14%)	44 (41%)	37 (35%)	26 (24%)	
Missing data	2 (0.2%)					7 (0.9%)				

*p* values were calculated using the *χ* ^2^ test.

AJCC, American Joint Committee on Cancer; ITBCC, International Tumor Budding Consensus Conference; MMR, mismatch repair; TIBI, Tumor Invasive Border Index.

### TIBI is associated with the composition of the tumor immune microenvironment

To study the composition of the immune microenvironment in detail, we utilized multiplex immunohistochemistry, combined with digital pathology in cohort 1. High‐TIBI tumors demonstrated lower densities of M1‐like macrophages and CD66B^+^ granulocytes, while low TIBI tumors showed lower densities of tryptase^+^ mast cells (*p* < 0.001) (supplementary material, Figure S2). These findings were also consistent in MMR proficient cases but not observed in MMR deficient cases. Together, these findings highlight distinct immune microenvironment features associated with TIBI.

### TIBI predicts survival independent of tumor budding

**Figure 1:**
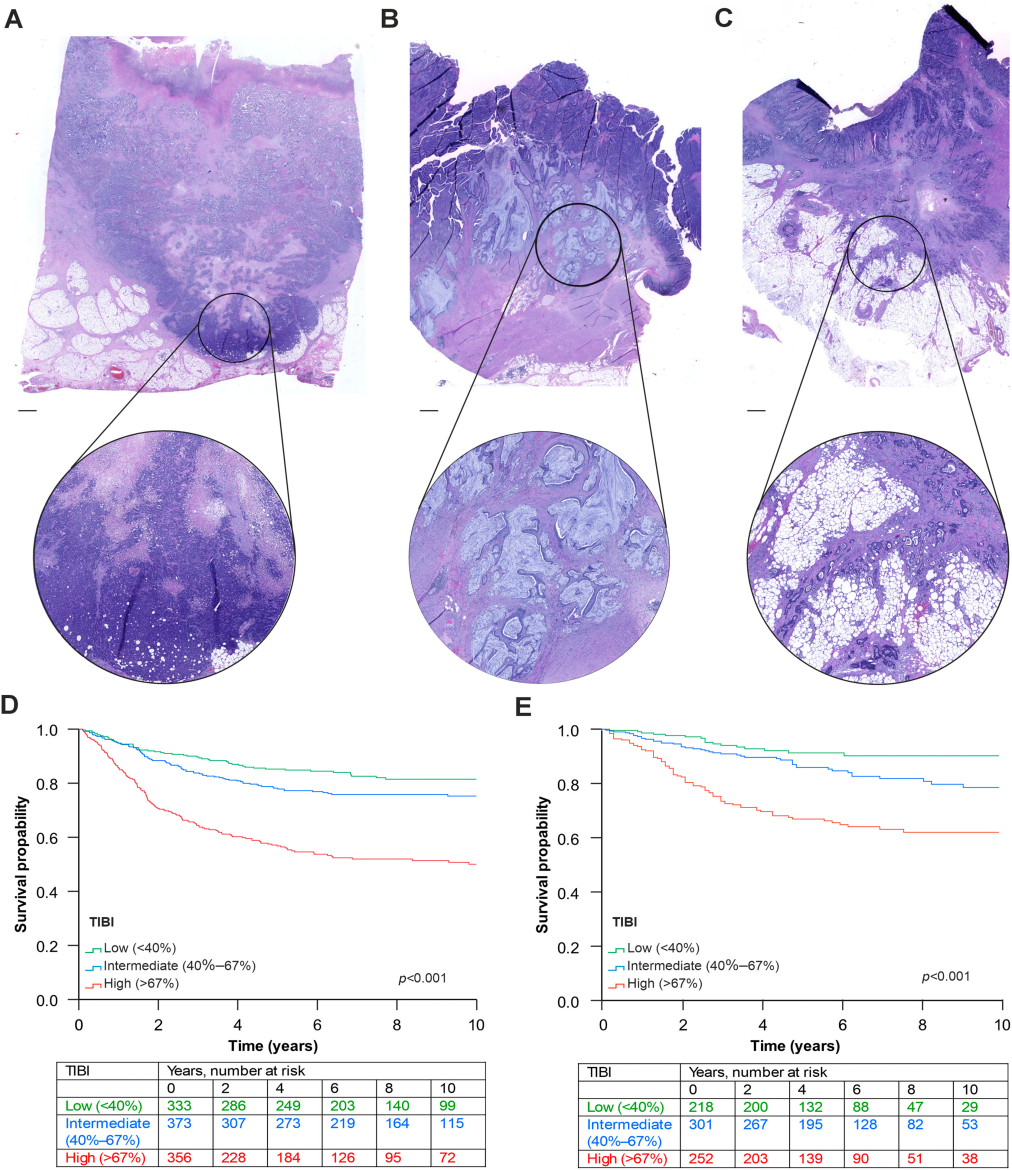
Examples of tumor samples with different Tumor Invasive Border Index (TIBI) categories and Kaplan–Meier survival analysis. (A) An example of a tumor with low TIBI (< 40%). (B) An example of a tumor with intermediate TIBI (40–67%). (C) An example of a tumor with high TIBI (> 67%). (D) Kaplan–Meier curves displaying cancer‐specific survival according to TIBI categories for cohort 1. (E) Kaplan–Meier curves displaying cancer‐specific survival according to TIBI categories for cohort 2. Scale bars, 1 mm.

Over the 10‐year follow‐up, there were 531 deaths (296 CRC) in cohort 1 and 264 deaths (146 CRC) in cohort 2. Kaplan–Meier analyses showed stepwise worse survival with increasing TIBI (Figure 1). In multivariable Cox regression models for cancer‐specific survival, TIBI was an independent prognostic factor [cohort 1: HRs for high (versus low) TIBI 1.52 (95% CI 1.07–2.17); cohort 2: HR 2.45 (95% CI 1.37–4.37)] (Table 2).

**Table 2:** Univariable and multivariable Cox regression models for cancer‐specific and overall survival according to TIBI classification.

		Colorectal cancer‐specific survival				Overall survival		
	No of cases	No of events	Univariable HR (95% CI)	Multivariable HR (95% CI)		No of events	Univariable HR (95% CI)	Multivariable HR (95% CI)
**Cohort 1**								
TIBI								
Low (< 40%)	333	54	1 (reference)	1 (reference)		147	1 (reference)	1 (reference)
Intermediate (40%–67%)	373	85	1.43 (1.02–2.01)	1.03 (0.72–1.47)		161	0.96 (0.78–1.18)	0.92 (0.73–1.17)
High (> 67%)	357	157	3.47 (2.54–4.72)	1.52 (1.07–2.17)		223	1.78 (1.47–2.16)	1.32 (1.04–1.68)
*p*_*trend*_			< 0.001	0.005			< 0.001	0.015
**Cohort 2**								
TIBI								
Low (< 40%)	218	20	1 (reference)	1 (reference)		58	1 (reference)	1 (reference)
Intermediate (40%–67%)	301	50	1.91 (1.09–3.33)	1.88 (1.04–3.41)		98	1.18 (0.89–1.64)	1.21 (0.86–1.71)
High (> 67%)	252	76	4.70 (2.79–7.91)	2.45 (1.37–4.37)		108	1.76 (1.28–2.42)	1.34 (0.94–1.92)
*p*_*trend*_			< 0.001	0.003			< 0.001	0.12

Multivariable Cox proportional hazards regression models were adjusted for sex, age (< 65, 65–75, > 75), year of operation (2000–2005, 2006–2010, 2011–2015, 2016–2020), tumor location (proximal colon, distal colon, rectum), AJCC T class (T1–2, T3–4), AJCC N class (N0, N1–2), AJCC M class (M0, M1), tumor grade (low grade, high grade), lymphovascular invasion (negative, positive), mismatch repair (MMR) status (proficient, deficient), *BRAF* status (wild‐type, mutant). Missing data on *BRAF* status (*n* = 1 in cohort 1, *n* = 7 in cohort 2) were included in the major category (*BRAF* wild‐type) to reduce the degrees of freedom.

*p*_trend_ values were calculated by using the three ordinal categories of TIBI as continuous variables in univariable and multivariable Cox proportional hazards regression models.

AJCC, American Joint Committee on Cancer; TIBI, Tumor Invasive Border Index.

Adjusting additionally for tumor budding, high TIBI remained significantly associated with worse survival (supplementary material, Table S1). The HR for high (versus low) TIBI was 1.43 (95% CI 1.00–2.06) in cohort 1 and 2.03 (95% CI 1.11–3.71) in cohort 2, whereas the corresponding HRs for high (versus low) tumor budding were 1.24 (95% CI 0.90–1.71) in cohort 1 and 1.67 (95% CI 1.08–2.58) in cohort 2, respectively. Full models for CSS and OS are provided in the supplementary material, Table S2. Subgroup analyses showed consistent associations with outcome across patient strata (supplementary material, Figure S6).

### High TIBI is associated with *TP53* and *KRAS* mutations, EMT‐associated genes, and downregulation of MYC signaling

To investigate molecular features underlying infiltrative tumor growth, TIBI was assessed in H&E‐stained sections from the TCGA cohort. There were 93 cases in the ‘low’, 145 in the ‘intermediate’, and 112 in the ‘high’ TIBI category.

**Figure 2:**
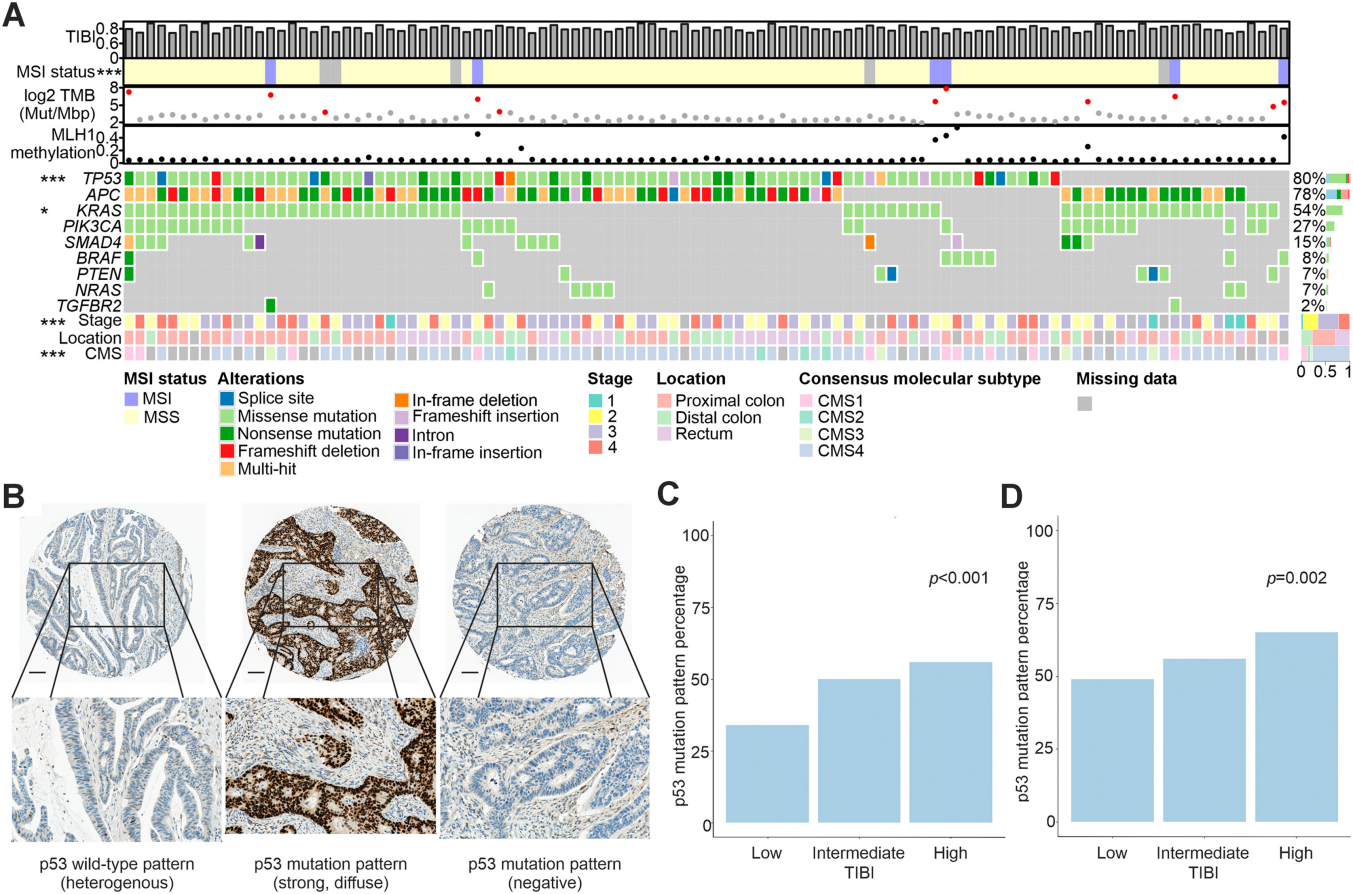
Mutational landscape of infiltrative colorectal cancer. (A) Heatmap of The Cancer Genome Atlas (TCGA) colorectal cancer cases with high Tumor Invasive Border Index (TIBI) (> 0.67) (*n* = 109), displaying mutations in common colorectal cancer associated genes alongside key clinical features. (B–D) Immunohistochemical analysis of TP53 expression in cohorts 1 (*n* = 967) and 2 (*n* = 760). (B) Representative examples of wild‐type and mutation expression patterns of TP53. (C, D) Bar charts demonstrating higher frequency of TP53 mutation pattern in higher TIBI categories in (C) cohort 1 and in (D) cohort 2. *p < 0.05; **p < 0.01; ***p < 0.001. *p* values are for the Fisher's test. Scale bars, 100 μm.

High‐TIBI tumors showed more frequent mutations of *TP53* (80% versus 54%, *p* < 0.001) and *KRAS* (54% versus 36%, *p* = 0.030) compared with other tumors (Figure 2A). The higher frequency of *TP53* alteration in infiltrative tumors was validated via immunohistochemistry (*p <* 0.001 in cohort 1, *p* = 0.002 in cohort 2) (Figure 2B–D). High‐TIBI tumors in the TCGA cohort were enriched for consensus molecular subtype 4 (CMS4) (60% versus 13%, *p* < 0.001) and high stage (stages 3–4, 65% versus 36%, *p* < 0.001).

Genomic Identification of Significant Targets in Cancer (GISTIC) analysis showed frequent chromosome copy‐number alterations in high‐TIBI tumors, most notably a gain at 13q12.2 (*p* = 0.02 compared with low to intermediate TIBI tumors) (supplementary material, Figure S7A). To further investigate structural alterations in this region, we performed Optical Genome Mapping on a subset (*n* = 35) of tumors from cohort 2 (supplementary material, Figure S7B,C). Although the sample size limited statistical power, there was a non‐significant trend towards increased 13q12.2 gains in high‐TIBI cases compared with low/intermediate‐TIBI cases (67% versus 40%, *p* = 0.176) (supplementary material, Figure S7D).

**Figure 3:**
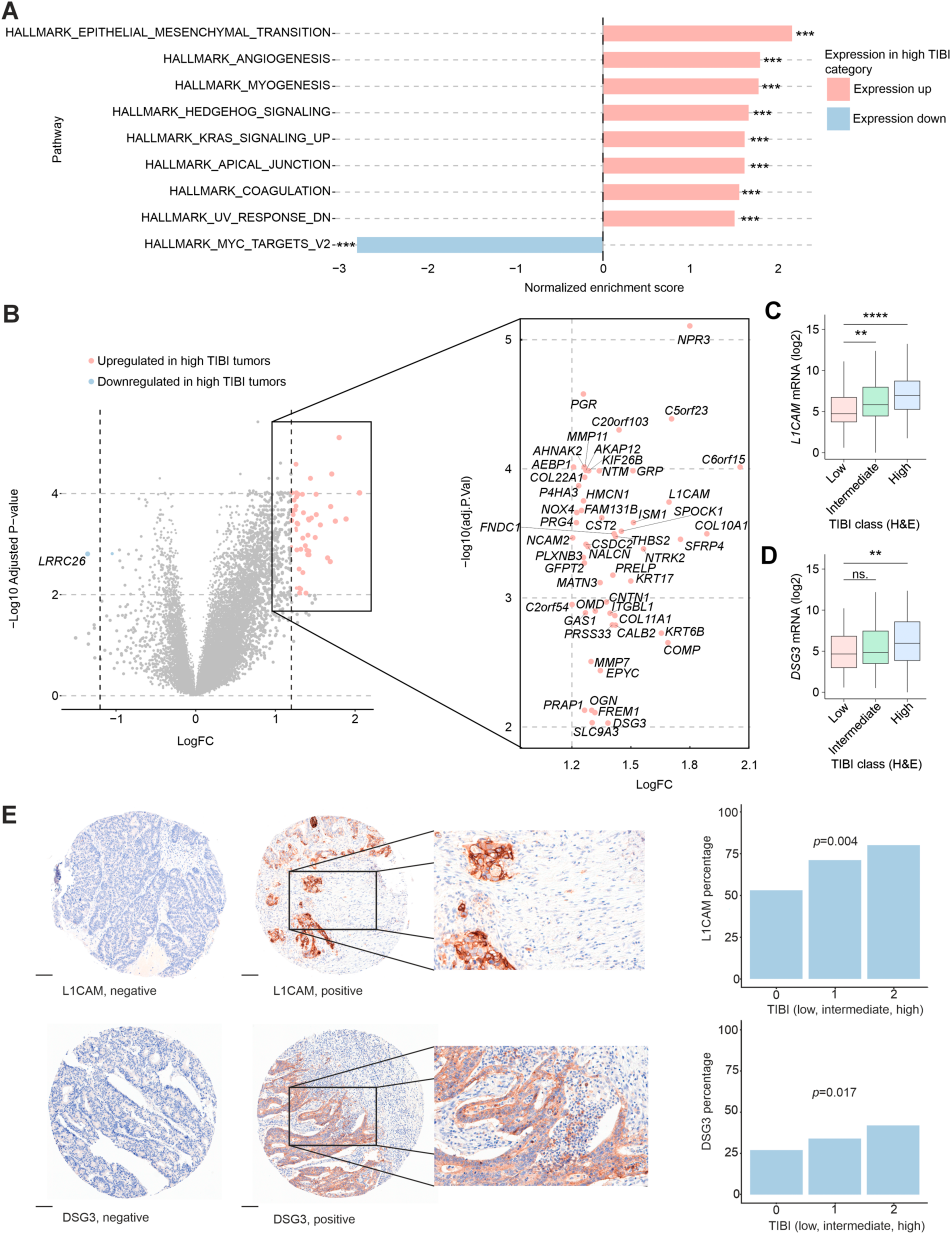
Gene expression patterns of infiltrative colorectal cancers. (A) Gene set enrichment analysis comparing high Tumor Invasive Border Index (TIBI) cases with low TIBI cases in The Cancer Genome Atlas (TCGA) cohort (*n* = 201). (B) Volcano plot in of the individual differentially expressed genes in the TCGA cohort. (C, D) Boxplots representing mRNA expression of (C) *L1CAM* and (D) *DSG3* in various TIBI classes. (E) Immunohistochemistry validation of L1CAM and DSG3 expression in cohort 2 (*n* = 760 for L1CAM and 759 for DSG3). Representative examples of positive and negative samples are shown, along with bar plots illustrating the frequency of L1CAM and DSG3 expression across TIBI categories. *p < 0.05, **p < 0.01, ***p < 0.001. *P‐*values are for the Mann–Whitney U test (panels C, D) or Fisher's test (panel E). Scale bars, 100 μm.

GSEA (Figure 3A) indicated that high‐TIBI tumors were associated with upregulation of the epithelial‐mesenchymal transition (EMT) pathway (*p* < 0.001). This aligned with the observation of a strong association with high TIBI and tumor budding in cohorts 1 and 2, as tumor budding is considered to represent EMT [38]. Other upregulated pathways in tumors with high TIBI included angiogenesis, myogenesis, KRAS signaling, and apical junction, among others, while MYC signaling was downregulated (*p* < 0.001). The downregulation of MYC expression was confirmed using immunohistochemistry in cohort 2 (*p* = 0.007) (supplementary material, Figure S8A). The individual differentially expressed genes between high and low TIBI categories are presented in Figure 3B; among these, L1CAM and DSG3 showed higher mRNA expression in high‐TIBI tumors (Figure 3C,D). From these genes, *L1CAM*, *DSG3*, and *KRT17* were selected for further examination based on their expected expression in tumor epithelium and availability of well‐validated antibodies. Immunohistochemistry demonstrated prominent L1CAM and DSG3 staining at the invasive front (supplementary material, Figure S9A,B) and higher expression in high‐TIBI tumors (L1CAM *p* < 0.001, DSG3 *p* = 0.004), while KRT17 did not show a significant difference (*p* = 0.288) (Figure 3E; supplementary material, Figure S8B).

We evaluated the prognostic associations of L1CAM and DSG3 in cohort 2. Kaplan–Meier analyses (supplementary material, Figure S9C,D) suggested a tendency towards worse survival in L1CAM and DSG3 positive tumors (log‐rank *p* = 0.070 and *p* = 0.082, respectively). Cox regression models (supplementary material, Table S3) showed a concordant direction of effect but the associations remained non‐significant.

### TIBI‐associated genes *L1CAM* and *DSG3* promote invasion in colorectal cancer cells *in vitro*

**Figure 4:**
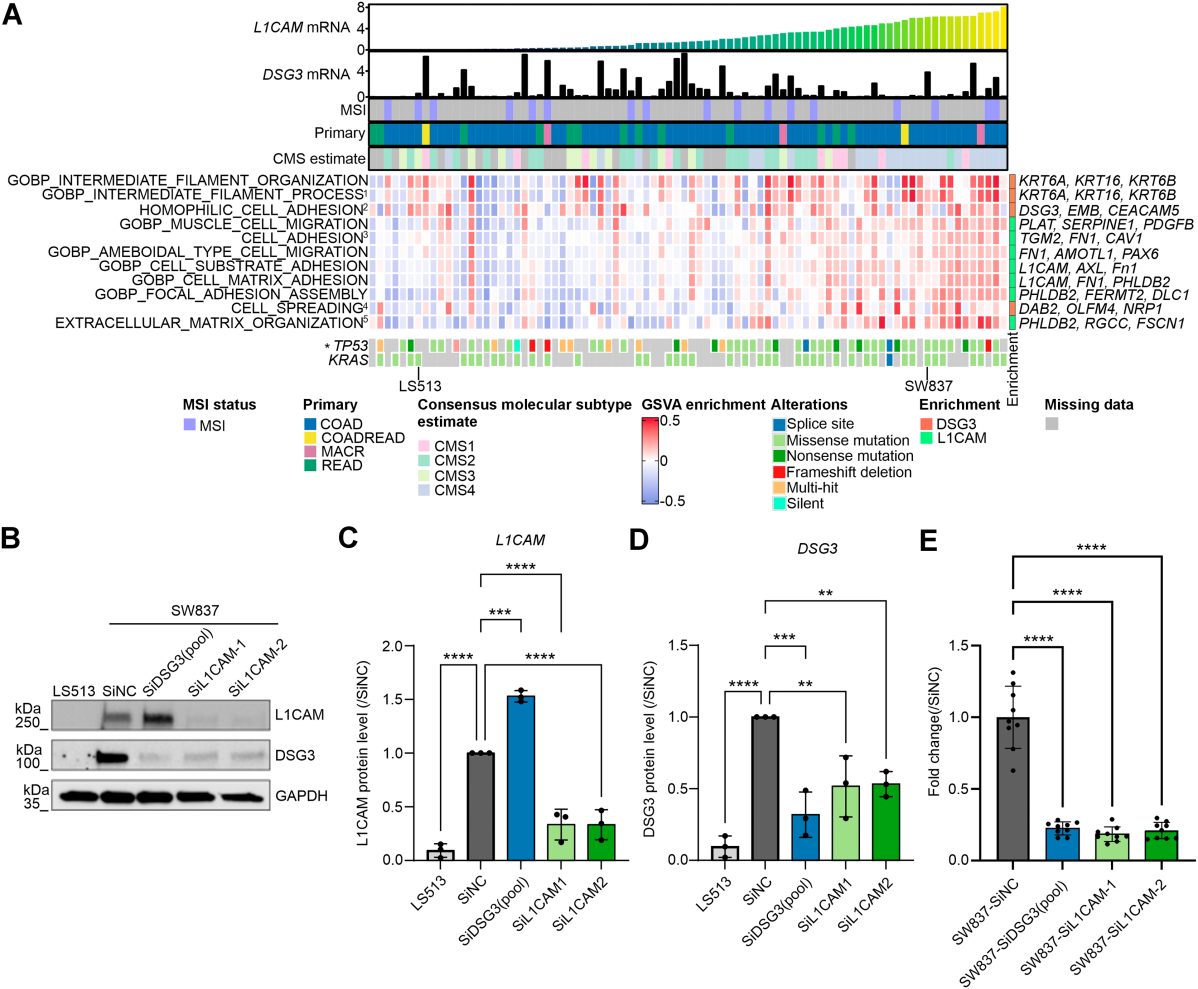
*In vitro* experiments on L1CAM and DSG3. (A) Heatmap of colorectal cancer cell lines from Cancer Cell Line Encyclopedia (CCLE), ordered by increasing *L1CAM* mRNA expression. (B) Western blotting showing L1CAM and DSG3 protein expression after L1CAM or DSG3 silencing in SW837 cells. SiNC‐transfected SW837 cells and untreated LS513 cells are shown as controls. (C) Quantification of L1CAM protein levels from western blotting. (D) Quantification of DSG3 protein levels from western blotting. (E) Matrigel invasion assay demonstrating the effect of L1CAM or DSG3 silencing on SW837 cell invasiveness. SiNC, non‐targeting control siRNA; siDSG3(pool), DSG3 siRNA pool; siL1CAM1/2, L1CAM siRNA 1/2. Superscript numbers in panel A indicate Gene Ontology Biological Process (GOBP) terms: ^1^,GOBP_ INTERMEDIATE_FILAMENT_ BASED_PROCESS; ^2^,GOBP_HOMOPHILIC_CELL_ADHESION_VIA_PLASMA_MEMBRANE_ADHESION_MOLECULES; ^3^,GOBP_POSITIVE_REGULATION_OF_CELL_ADHESION; ^4^,GOBP_POSITIVE_REGULATION_OF_SUBSTRATE_ADHESION_DEPENDENT_CELL_SPREADING; ^5^, GOBP_POSITIVE_REGULATION_OF_EXTRACELLULAR_MATRIX_ORGANIZATION. *p < 0.05, **p < 0.01, ***p < 0.001, ****p < 0.0001.

Building on our findings linking specific genes to infiltrative growth in CRC, we explored the potential functional roles of the TIBI‐associated genes *L1CAM* and *DSG3*. We first analyzed their expression and related biological processes in CRC cell lines in the Cancer Cell Line Encyclopedia (CCLE) dataset. *L1CAM* expression was significantly associated with *TP53* mutation (*p* = 0.018), CMS4 (*p* < 0.001) (supplementary material, Figure S10B), and enrichment in pathways related to extracellular matrix (ECM) remodeling, matrix adhesion, and cell migration, consistent with findings from tumor tissue (Figure 4A). Interestingly, while *DSG3* was not as strongly correlated with ECM modification and cell migration (Figure 4A), its expression was associated with squamous differentiation and mechanical resilience, as indicated by co‐expression with *KRT6A*, *KRT6B*, and *KRT16*.

*L1CAM* expression correlated strongly with *TGM2*, an enzyme known to crosslink matrix components, with structural ECM components *FN1* and *COL13A1* (supplementary material, Figure S10A), and with FN receptor *ITGA5* (supplementary material, Figure S10C). Correlations were also observed with *AXL* tyrosine kinase, an established regulator of breast cancer invasion and metastasis, and additional EMT markers, including *DLC1* [39, 40].

As these genes correlated strongly with invasive features in CRC cell lines, we tested their effects on cancer invasion *in vitro*. Based on baseline expression, SW837 cells (high *L1CAM*/*DSG3*) were selected for functional characterization, while LS513 cells (low *L1CAM*/*DSG3*) were included as a negative control (Figure 4A). In SW837 cells, *DSG3* silencing upregulated *L1CAM*, while *L1CAM* silencing reduced both *L1CAM* and *DSG3* (Figure 4B–D). Independent knockdown of *L1CAM* or *DSG3* reduced cell invasion by 75% in Matrigel‐coated transwells versus controls (Figure 4E), without affecting cell proliferation (supplementary material, Figure S10D). These experiments functionally link TIBI‐associated epithelial markers to invasive capacity, supporting the biological relevance of TIBI.

### Transcriptomic signature of TIBI‐associated genes is linked with infiltrative tumors and predicts similar clinicopathological attributes in gastric adenocarcinoma

We derived a TIBI‐t score (supplementary material, Table S4) from genes differentially expressed between TIBI categories in TCGA COAD/READ. Across TCGA, TIBI‐t was highest in generally infiltrative cancers such as pancreatic, breast, and esophageal carcinomas and lowest in tumor types with pushing borders such as hepatocellular carcinoma, adrenocortical carcinoma, and renal cell carcinomas (supplementary material, Figure S11A, Table S5).

In TCGA gastric adenocarcinomas, we visually scored H&E sections for tumors in the top/bottom 4% of TIBI‐t (supplementary material, Figure S11B,C). Among these tumors, high TIBI was strongly associated with high TIBI‐t (supplementary material, Figure S11D). High TIBI‐t showed EMT enrichment and downregulation of MYC targets (supplementary material, Figure S11E). Unlike CRC, high TIBI‐t in STAD associated with lower *TP53* mutation odds (*p* = 0.009). However, high TIBI‐t in STAD was associated with diffuse histology and E‐cadherin loss (*p* < 0.001), and *TP53* mutation frequency is generally lower in diffuse gastric cancer. Prognostically, TIBI‐t independently predicted worse outcomes in STAD after adjusting for age and stage: disease‐specific survival, HR = 1.18, 95% CI 1.05–1.37; progression‐free interval, HR = 1.19, 95% CI 1.04–1.29; disease‐free interval, HR = 1.40, 95% CI 1.08–1.58 (supplementary material, Figure S11F–H).

## Discussion

Our study introduces the TIBI as a reproducible method for classifying tumor border configuration. High TIBI, reflecting an infiltrative tumor border configuration, was strongly associated with adverse tumor characteristics, including advanced stage, lymphovascular invasion, MMR proficiency, high tumor budding, and *TP53* mutation. Importantly, high TIBI independently predicted shorter CSS across two large cohorts, even after adjusting for conventional prognostic factors and tumor budding. These findings position TIBI as a valuable tool for stratifying patients with CRC. By linking TIBI to molecular features such as an EMT signature and distinct immune microenvironment profiles, our study provides a framework for understanding the biological underpinnings of infiltrative tumor growth in CRC.

Various histomorphological biomarkers have emerged in CRC over the past decades, including tumor budding and tumor‐stroma ratio. Tumor‐stroma ratio shares methodological similarity with TIBI in that both evaluate tissue composition on routine H&E sections. However, TIBI is assessed in a larger (diameter 4 mm), standardized hotspot anchored to the deepest invasive margin and incorporates fibrous stroma and adipose tissue at the invasive interface, thereby focusing on tumor border configuration and infiltrative growth. By contrast, tumor‐stroma ratio is scored in a selected, smaller microscopic field (10× field of view corresponding to diameter ~ 2 mm) enriched for stroma and reflects stromal abundance relative to tumor epithelium [41]. Despite overlap in underlying biology, including links to EMT [42], these differences in region selection and tissue components mean that TIBI is designed to capture invasive‐front configuration rather than stromal richness alone.

We compared TIBI with tumor budding, a well‐established prognostic marker in CRC. TIBI demonstrated high inter‐rater agreement, comparable to or better than tumor budding. In our survival analyses, the prognostic significance of TIBI remained statistically significant even after adjusting for tumor budding, underscoring the independent prognostic value of TIBI. Our findings suggest that TIBI may support clinical risk stratification. This may be particularly useful in stage II CRC, where adjuvant treatment decisions are often challenging. As an H&E‐based metric, TIBI could complement established or emerging high‐risk features (such as tumor budding, poorly differentiated clusters, tumor deposits, or stromal maturity) to identify patients who may warrant consideration of adjuvant therapy and closer surveillance. Because treatment data were incomplete, TIBI should be interpreted here as a prognostic marker, and any treatment‐predictive utility requires prospective validation.

Our findings align biologically with prior observations that infiltrative tumor growth marks aggressive disease. In our data, high TIBI co‐occurred with high tumor budding, a feature widely viewed as a histologic surrogate of EMT and linked to invasion and adverse outcome [11, 38]. Molecular analyses in TCGA further supported this biology: high‐TIBI tumors were enriched for CMS4 and EMT, angiogenesis, and KRAS signaling. The genetic features were also consistent with canonical CRC pathways, as high‐TIBI tumors showed more frequent *TP53* and *KRAS* alterations characteristic of chromosomal instability and MMR proficient carcinogenesis [43].

The tumor‐immune contexture also differed by TIBI. Using three multiplex immunohistochemistry assays, we observed lower densities of M1‐like macrophages and CD66B^+^ granulocytes in infiltrative tumors. Compared with previous studies [16, 18], our approach enabled more detailed cell phenotyping by multiplex immunohistochemistry coupled with digital pathology. For example, macrophage phenotypes (M1‐like and M2‐like) were classified based on the expression of four polarization markers, given the absence of a single definitive marker [44]. However, the observed associations were relatively modest, suggesting that the intrinsic, genetic properties of the tumor may play a more dominant role in shaping the tumor border configuration than the host immune response.

At the single‐gene level, *L1CAM* and *DSG3* showed higher expression at the invasive front in high‐TIBI tumors. We emphasize that infiltrative border morphology likely reflects heterogeneous biology, which is consistent with the gradual protein‐level differences observed by immunohistochemistry. Our multi‐omic analyses therefore point to candidate genes and pathways that may contribute to infiltrative growth in subsets of tumors. DSG3, a desmosomal adhesion protein has emerging relevance in multiple cancers though its mechanisms remain incompletely defined [45, 46, 47]. Consistent with this, *DSG3* was highly expressed at the border of high‐TIBI tumors, and its depletion in a model cell line significantly reduced invasion. DSG3 may mediate these changes through various pathways, including the upregulation of matrix metalloproteinases or by influencing cell polarity [48]. L1CAM, originally characterized in neural tissue, is overexpressed in several cancers, including CRC [49], and can act as a ligand for fibronectin binding integrins [39]. In our data, *L1CAM* expression correlated with *FN* and its receptor *ITGA5*, suggesting a possible functional link between these factors. Additionally, L1CAM is a positive regulator of β‐catenin signaling and promotes stemness, invasion, motility, and metastasis initiation, all important drivers of EMT and infiltrating tumor growth [49, 50, 51]. Functionally, *L1CAM* knockdown inhibited invasion *in vitro* and concomitantly reduced DSG3 protein, indicating a plausible crosstalk between these two pro‐invasive proteins, whereas *DSG3* knockdown also impaired invasion. Collectively, these findings implicate altered adhesion and EMT programs in high‐TIBI tumors and illustrate how TIBI can bridge diagnostic morphology with mechanistic hypotheses and potential invasion‐related molecular targets across solid cancers.

We also derived a gene expression signature (TIBI‐t) that captured the TIBI‐associated program and generalized beyond CRC. Across TCGA tumor types, TIBI‐t was highest in malignancies known for infiltrative growth and lowest in cancers that characteristically have pushing borders. We extended TIBI‐t analyses to gastric adenocarcinoma, as it represents a related gastrointestinal adenocarcinoma and gastric wall layers provide an anatomic reference that enables consistent identification of the point of deepest invasion. TCGA STAD further provided adequate numbers of evaluable cases for this analysis. In gastric adenocarcinoma, high TIBI‐t aligned with histologic infiltrative growth on H&E, EMT enrichment, and loss of E‐cadherin, a prominent molecular feature of diffuse growth [52]. It also independently predicted worse outcome. These data expand the prognostic potential of TIBI beyond CRC and establish a robust mRNA surrogate for assessing TIBI from transcriptomic data.

Our study has limitations. First, immune cell densities were defined using TMAs, which sample only a fraction of each tumor. However, previous studies have shown that TMAs provide a reasonable approximation of immune cell counts across the entire tumor [20]. Second, visual estimation of areal fractions for TIBI, although intentionally simple, may introduce interobserver variability; the detailed manual and our inter‐rater data mitigate this concern. Third, incomplete data on adjuvant treatments limited our ability to evaluate the predictive value of TIBI for specific therapies. Fourth, TNM staging criteria have evolved over the cohort collection period, and the stages assigned reflect the TNM edition used at the time of diagnosis. These changes were incremental refinements within an anatomy‐based system rather than major conceptual changes, and as stage was used for adjustment rather than as a primary endpoint, this is unlikely to have materially influenced our conclusions. Fifth, the *in vitro* experiments were performed in a single CRC cell line and should be interpreted as supportive and hypothesis‐generating; validation across a broader panel of CRC models, including both MMR proficient and deficient backgrounds, will be important in future work. Sixth, not all immunohistochemistry assays were available in both cohorts. The consistency between TCGA transcriptomic associations and protein‐level associations supports the L1CAM, DSG3, and MYC findings, although replication of the full immunohistochemistry panel across cohorts will be valuable in future studies. Strengths include two large, well‐annotated cohorts with long follow‐up, orthogonal validation in TCGA, quantitative multiplex immune profiling, and functional experiments that support causality for TIBI‐associated genes.

In conclusion, TIBI is a novel and robust method for assessing tumor border configuration with reproducibility and prognostic significance comparable to established histomorphological factors, such as tumor budding. Our study provided insights into the tumor properties, immune cell landscapes, and molecular features underlying infiltrative tumor border configuration. These findings support the integration of TIBI into routine diagnostic workflows for assessing tumor border configuration and highlight its potential role in informing personalized treatment for CRC patients.

## Author contributions statement

AK, JH, AT and JPV were responsible for conceptualization. AK, PS, VKÄ, HK, MK, VVT, TM, HE, OS, MA, OH, E‐VW, JB, MJM, AT, JI and JPV contributed to data curation. AK, JH, HL and JPV performed formal analysis. AK, PS, MJM and JPV were responsible for funding acquisition. JH, HL, PS, VKÄ, HK, MK, VVT, TM, V‐MP, HE, OS, NP, MA, OH, E‐VW, TTM, OL, JaS, JR, SM, JuS, TR, TTS, JB, J‐PM, MJM, AT, JI and JPV contributed to investigation. AK, JH, HL, JI and JPV were responsible for methodology. AT, JI and JPV contributed to supervision. AK, JH, HL and JPV contributed to visualization. AK wrote the original draft. JH, HL, PS, VKÄ, HK, MK, VVT, TM, V‐MP, HE, OS, NP, MA, OH, E‐VW, TTM, OL, JaS, JR, SM, JuS, TR, TTS, JB, J‐PM, MJM, AT, JI and JPV contributed to review and editing of the manuscript. All authors approved the final version of the manuscript.

## Supporting information


**Figure S1.** Flowchart of patient selection
**Figure S2.** Immune cell densities by Tumor Invasive Border Index (TIBI) categories
**Figure S3.** Expression of Tumor Invasive Border Index (TIBI)‐associated genes in single cell RNA‐seq data
**Figure S4.** Establishing criteria for Tumor Invasive Border Index (TIBI)
**Figure S5.** Inter‐rater assessment of Tumor Invasive Border Index (TIBI)
**Figure S6.** Prognostic impact of Tumor Invasive Border Index (TIBI) across various patient subgroups
**Figure S7.** Chromosome copy‐number variation related to infiltrative growth pattern
**Figure S8.** Associations between Tumor Invasive Border Index (TIBI) and tumor molecular features
**Figure S9.** L1CAM and DSG3 immunohistochemistry at the tumor margin
**Figure S10.** Additional data from the Cancer Cell Line Encyclopedia (CCLE) dataset and cell culture experiments
**Figure S11.** Pan‐cancer and gastric cancer analysis of transcriptomic Tumor Invasive Border Index (TIBI‐t) signature
**Table S1.** Cox regression models for cancer‐specific survival according to ITCBB tumor budding classification and tumor border TIBI configuration
**Table S2.** Multivariable Cox regression models of cancer‐specific survival and overall survival according to TIBI levels and other covariates
**Table S3.** Univariable and multivariable Cox regression models for cancer‐specific and overall survival according to L1CAM and DSG3 expression in cohort 2
**Table S4.** Genes chosen for TIBI‐t score and their weight in the score
**Table S5.** TCGA cohort abbreviations shown in the supplementary material, Figure S11

**File S1.** Tumor Invasive Border Index (TIBI) categorization of H&E sections available for The Cancer Genome Atlas (TCGA) COAD and READ cases (provided as a separate Excel file)

**File S2.** Tumor Invasive Border Index (TIBI) evaluation manual (provided as a separate Word document)

**File S3.** Tumor Invasive Border Index (TIBI) categorization of H&E sections available for bottom/top 4% of The Cancer Genome Atlas (TCGA) STAD cases, ranked by TIBI‐t (provided as a separate Excel file)

## Data Availability

The human colon cancer atlas single‐cell RNAseq data for 62 colon cancer cases, as well as the associated metadata, were downloaded from GEO accession number GSE178341 (https://www.ncbi.nlm.nih.gov/geo/query/acc.cgi?acc=GSE178341). TCGA data were downloaded from public repositories, including GDC Data Portal (https://gdc.cancer.gov/about-data/publications/pancanatlas) and cBioPortal (https://www.cbioportal.org/). Cancer Cell Line Encyclopedia data for somatic mutations and gene expression were downloaded from DepMap (https://depmap.org/portal/). Other data generated and/or analyzed during this study are not publicly available. Access to the data requires approval from relevant ethics committees and/or biobanks. Further information, including procedures to obtain and access data from Finnish Biobanks, are available from Finnish Biobank Cooperative – FINBB at https://finbb.fi/en/fingenious-service.
