# Supplementary material for: Tumor Invasive Border Index (TIBI) in colorectal cancer: linking infiltrative morphology to molecular insights
Source: J Pathol. 2026 Jun 18;270(1):114–28. doi: 10.1002/path.70087 (PMC13431732; doi:10.1002/path.70087)
Supplement: Supplementary file 1 — Figure S1. Flowchart of patient selection Figure S2. Immune cell densities by Tumor Invasive Border Index (TIBI) categories Figure S3. Expression of Tumor Invasive Border Index (TIBI)‐associated genes in single cell RNA‐seq data Figure S4. Establishing criteria for Tumor Invasive Border Index (TIBI) Figure S5. Inter‐rater assessment of Tumor Invasive Border Index (TIBI) Figure S6. Prognostic impact of Tumor Invasive Border Index (TIBI) across various patient subgroups Figure S7. Chromosome copy‐number variation related to infiltrative growth pattern Figure S8. Associations between Tumor Invasive Border Index (TIBI) and tumor molecular features Figure S9. L1CAM and DSG3 immunohistochemistry at the tumor margin Figure S10. Additional data from the Cancer Cell Line Encyclopedia (CCLE) dataset and cell culture experiments Figure S11. Pan‐cancer and gastric cancer analysis of transcriptomic Tumor Invasive Border Index (TIBI‐t) signature Table S1. Cox regression models for cancer‐specific survival according to ITCBB tumor budding classification and tumor border TIBI configuration Table S2. Multivariable Cox regression models of cancer‐specific survival and overall survival according to TIBI levels and other covariates Table S3. Univariable and multivariable Cox regression models for cancer‐specific and overall survival according to L1CAM and DSG3 expression in cohort 2 Table S4. Genes chosen for TIBI‐t score and their weight in the score Table S5. TCGA cohort abbreviations shown in the supplementary material, Figure S11 [file PATH-270-114-s002.docx]

**Tumor Invasive Border Index (TIBI) in colorectal cancer: linking infiltrative morphology to molecular insights**

A Kehusmaa, J Härkönen, H Li *et al. J Pathol* <https://doi.org/10.1002/path.70087>

**Supplementary Figures S1–S11**

**Supplementary Tables S1–S5**

**Supplementary Files S1 and S3 are provided as separate Excel files**

**Supplementary File S2 is provided as a separate Word document**


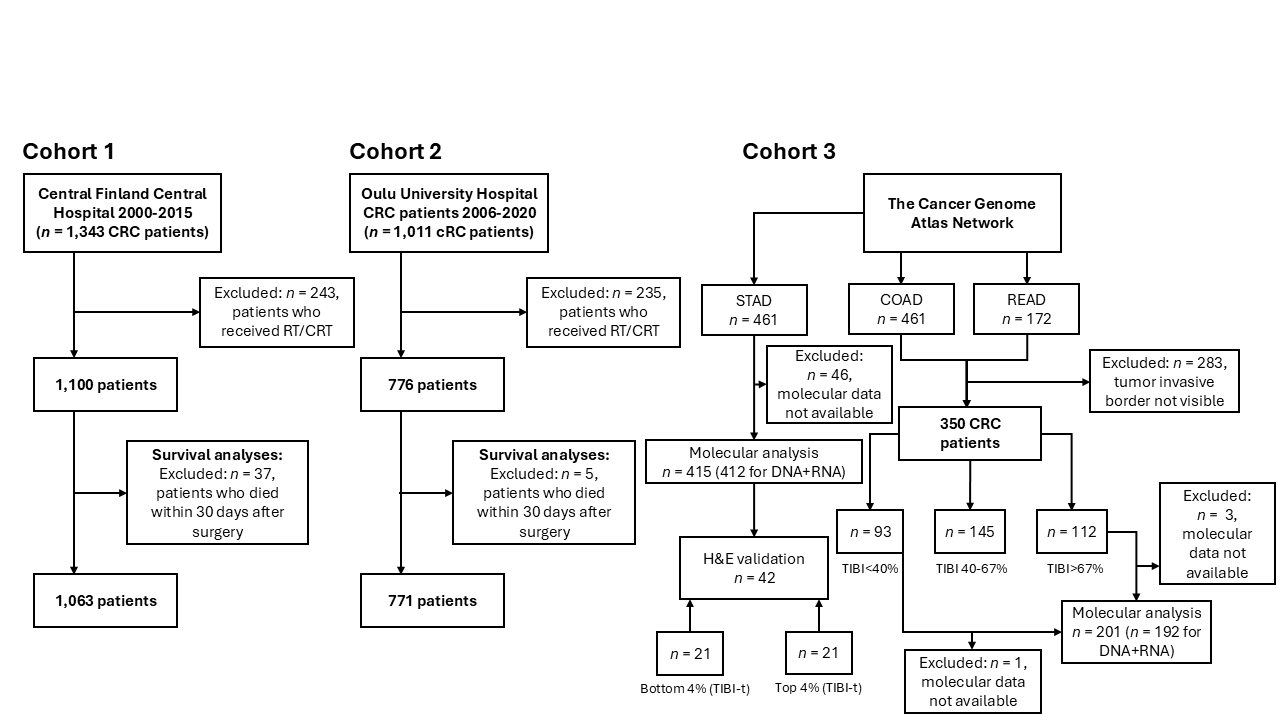


**Figure S1**. **Flowchart of patient selection**. Flowchart illustrating the inclusion criteria, patient exclusion process, and final patient numbers for the three study cohorts. COAD, colon adenocarcinoma; CRC, colorectal cancer; READ, rectal adenocarcinoma; RT/CRT, radiotherapy/chemoradiotherapy; TIBI, Tumor Invasive Border Index.


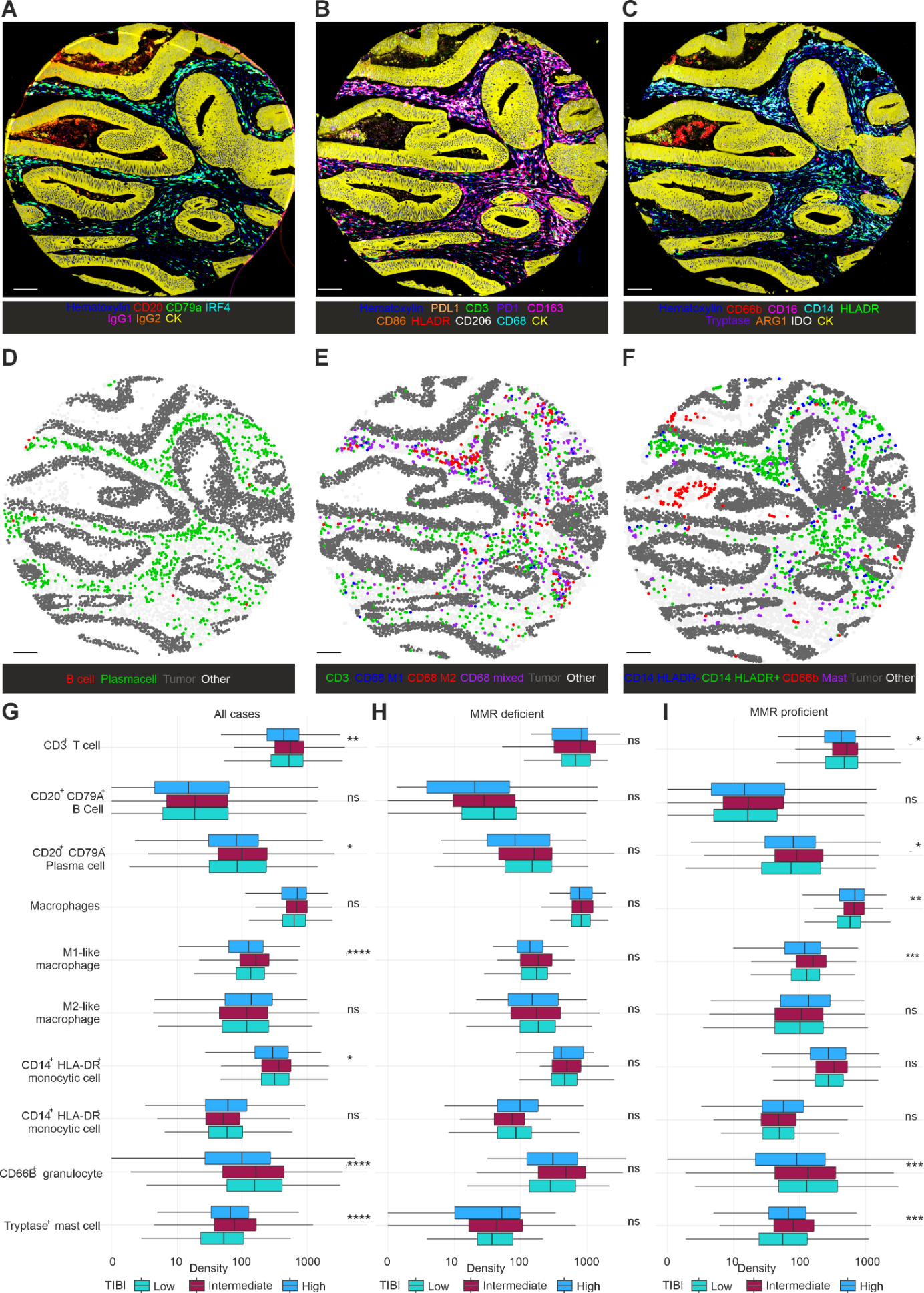


**Figure S2**. **Immune cell densities by Tumor Invasive Border Index (TIBI) categories**. (A–F) Representative examples of three multiplex immunohistochemistry assays, with panels (A–C) showing multiplex immunohistochemistry images, and panels (D,E) respective cell maps. (G–I) Boxplots of the distribution of immune cell densities across TIBI categories among (G) all cases, (H) mismatch repair (MMR) deficient cases, and (I) MMR proficient cases. The analyses were based on cohort 1: *n* = 1,065 for CD3^+^ T cells, macrophages, M1-like macrophages, and M2-like macrophages; *n* = 1,045 for CD14^+^HLA-DR^+^ mature monocytic cells, CD14^+^HLA-DR^-^ immature monocytic cells, CD66B^+^ granulocytes, and tryptase^+^ mast cells; *n* = 1,070 for CD20^+^CD79A^+^ B cells and CD20^-^CD79A^+^ plasma cells. **p* < 0.05, ***p* < 0.01, ****p* < 0.001‚ *****p* < 0.0001. *P-*values are for the Kruskal–Wallis test. Scale bars, 100 µm.


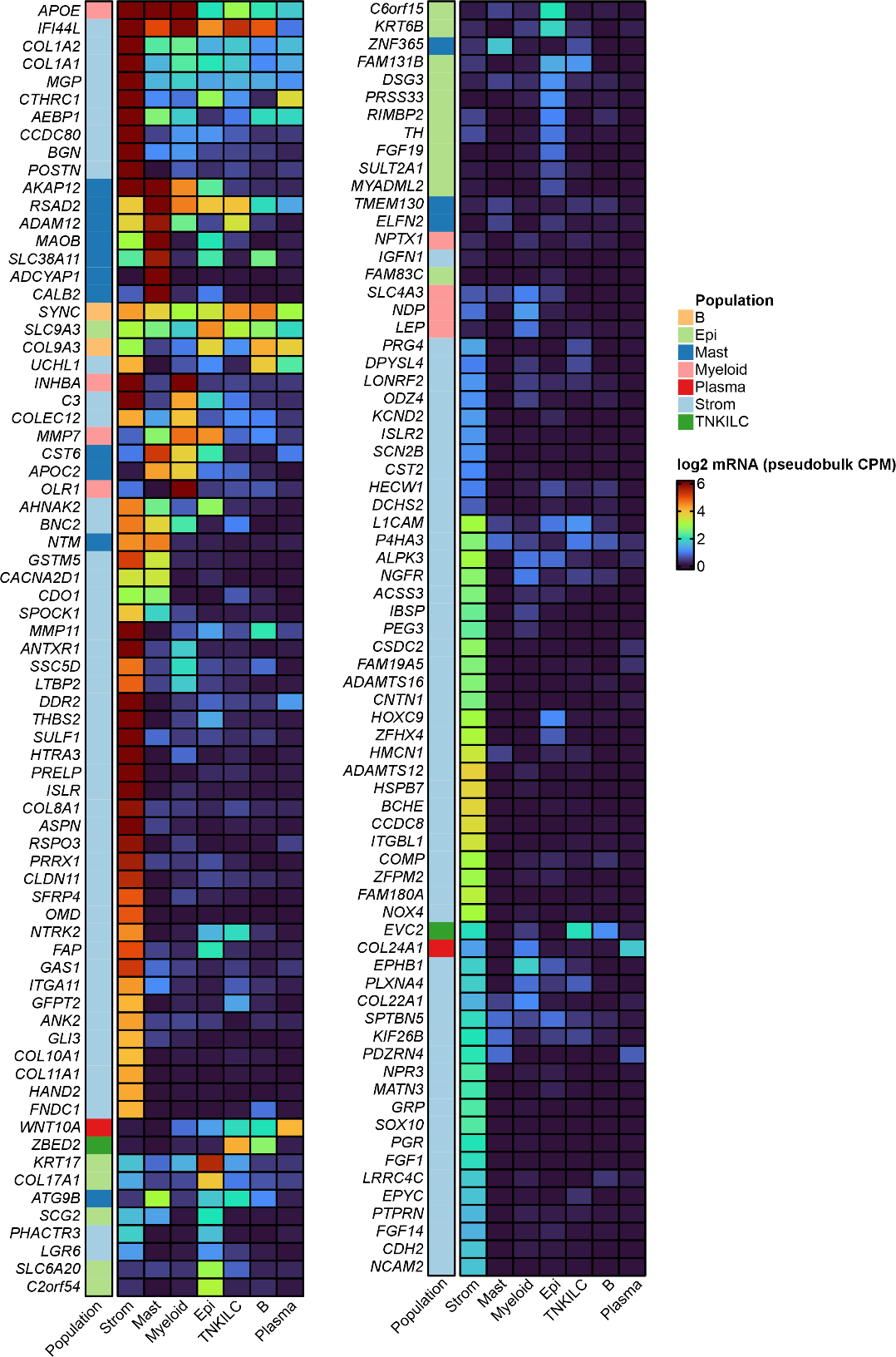


**Figure S3. Expression of Tumor Invasive Border Index (TIBI)-associated genes in single cell RNA-seq data**. The heatmap illustrates the expression patterns of the most differentially expressed genes identified in TCGA colorectal cancer cases across various cell populations from single cell RNA-seq data of 62 tumors. The cell population with the highest expression is highlighted in the first column. Genes most prominently expressed in stromal cells were used to derive the TIBI transcriptional signature (TIBI-t).


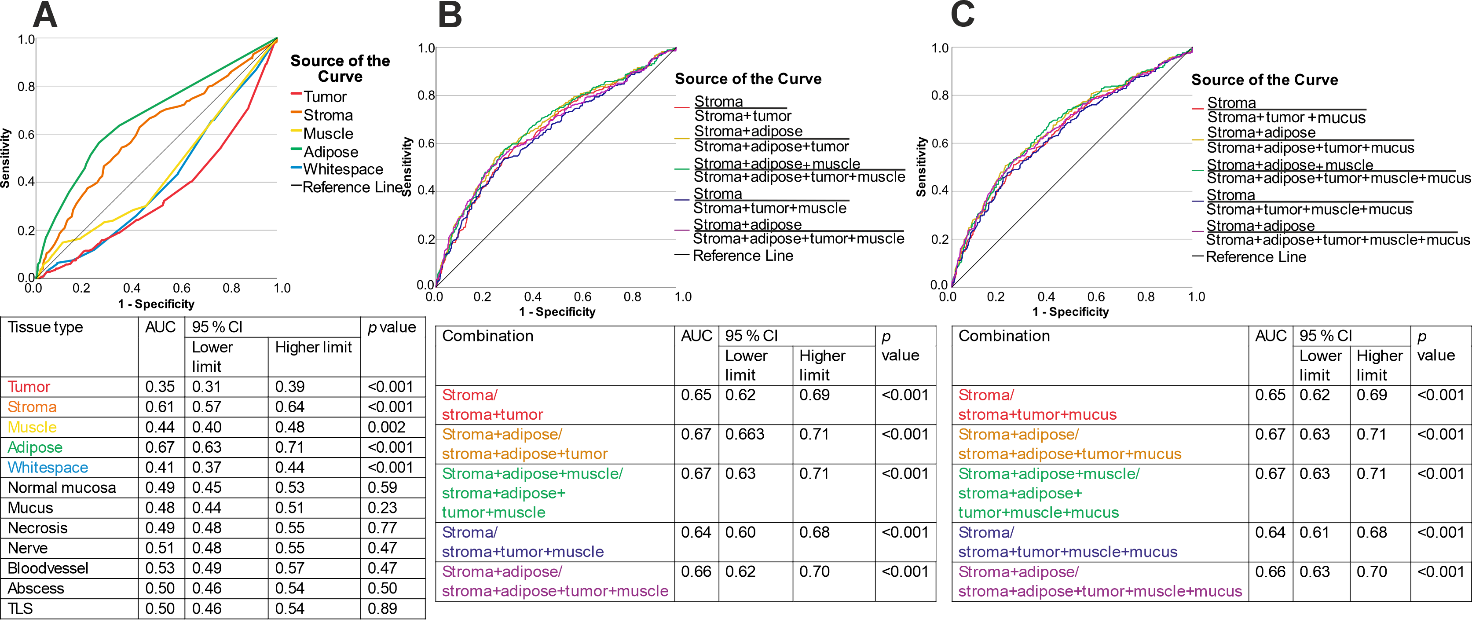


**Figure S4.** **Establishing criteria for Tumor Invasive Border Index (TIBI)**. Receiver operating characteristics (ROC) curves represent (A) the capacity of various tissue types and (B,C) different combinations of tissue types within a 4 mm diameter hotspot located at the deepest point of tumor invasion in classifying cancer-specific mortality in cohort 1. In panel (A), only the ROC curves with statistically significant area under the curve (AUC) are shown. Panel (B) represents the prognostic potential of potential combinations that do not include mucus, while mucus is included in panel (C). The final TIBI combination was chosen as one that had the highest AUC, while remaining easy to estimate. In particular, mucus was included in the model to facilitate the classification of mucinous tumors. TLS, tertiary lymphoid structure.


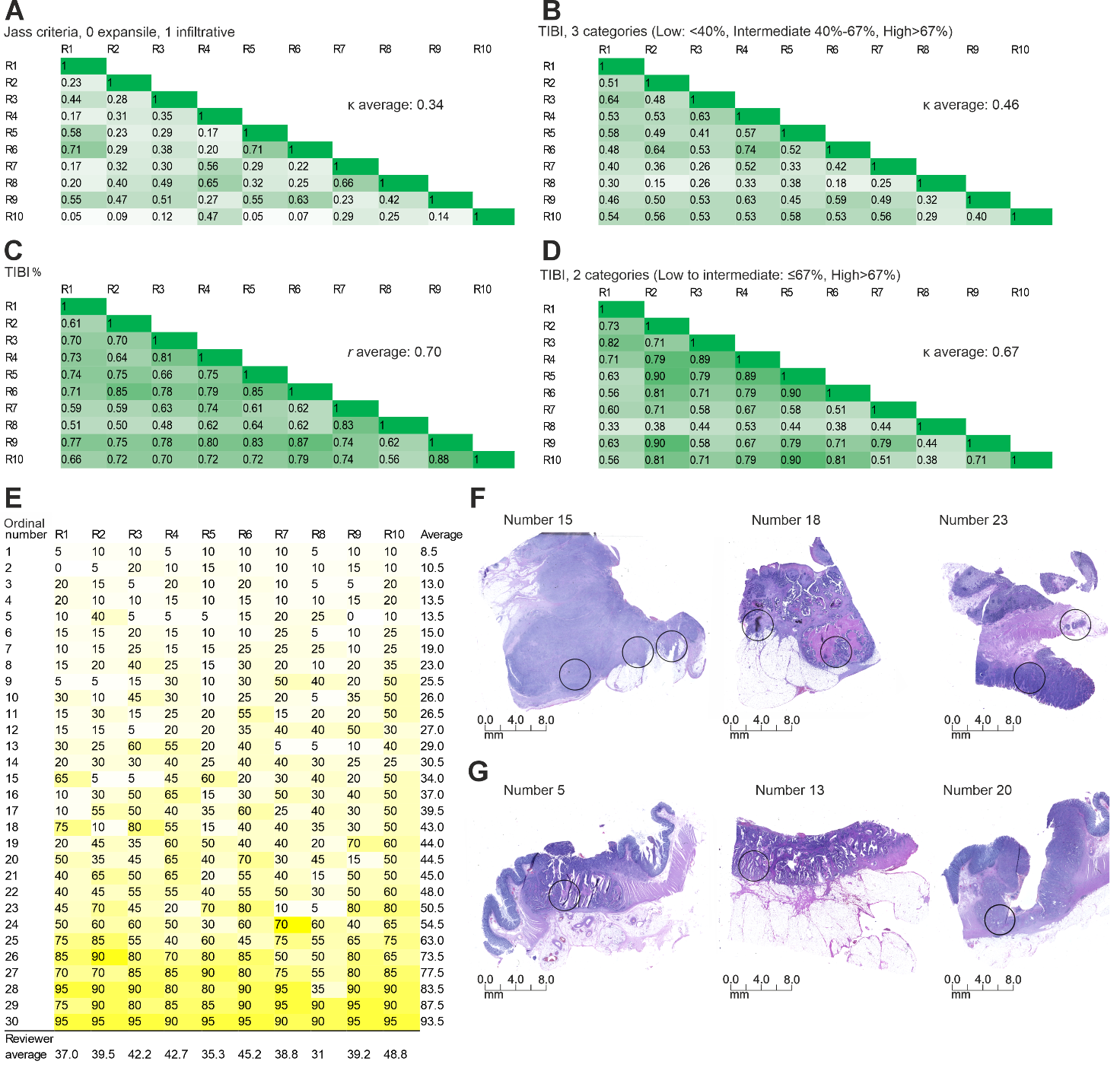


**Figure S5**. **Inter-rater assessment of Tumor Invasive Border Index (TIBI)**. (A) Inter-rater agreement kappa (κ) values of Jass criteria. (B–D) Inter-rater agreement for TIBI. (B) TIBI in three categories (κ coefficients). (C) TIBI as a continuous variable (Spearman’s correlation coefficients). (D) TIBI as a binary variable (κ coefficients). (E). TIBI percentage estimates of all raters in each sample, arranged by the average of the raters. (F) Example cases illustrating discrepancies due to hotspot location selection. (G) Example cases with differing percentage estimates despite a similar hotspot selection among raters.

**
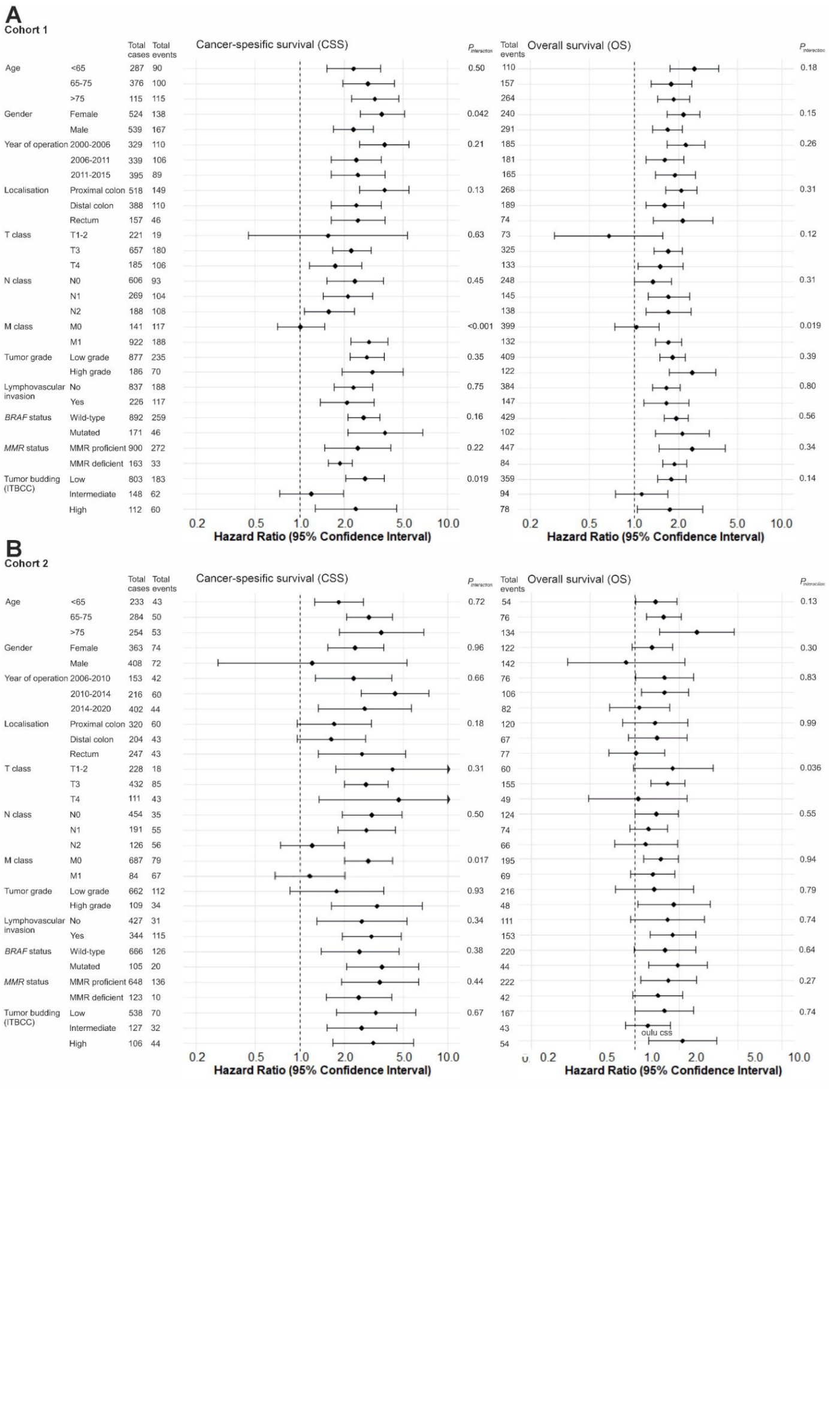
**

**Figure S6. Prognostic impact of Tumor Invasive Border Index (TIBI) across various patient subgroups.** Forest plots present hazard ratios and 95% confidence intervals for high (versus low/intermediate) TIBI in various patient subgroups in (A) cohort 1 and (B) cohort 2.

CSS, cancer specific survival; OS, overall survival; MMR, mismatch repair; ITBCC, Internation Tumor Budding Consensus Conference. *P*_interaction_ values were calculated using the Wald test for the cross product of the TIBI (high versus low/intermediate) and the variable of interest in the Cox regression model.


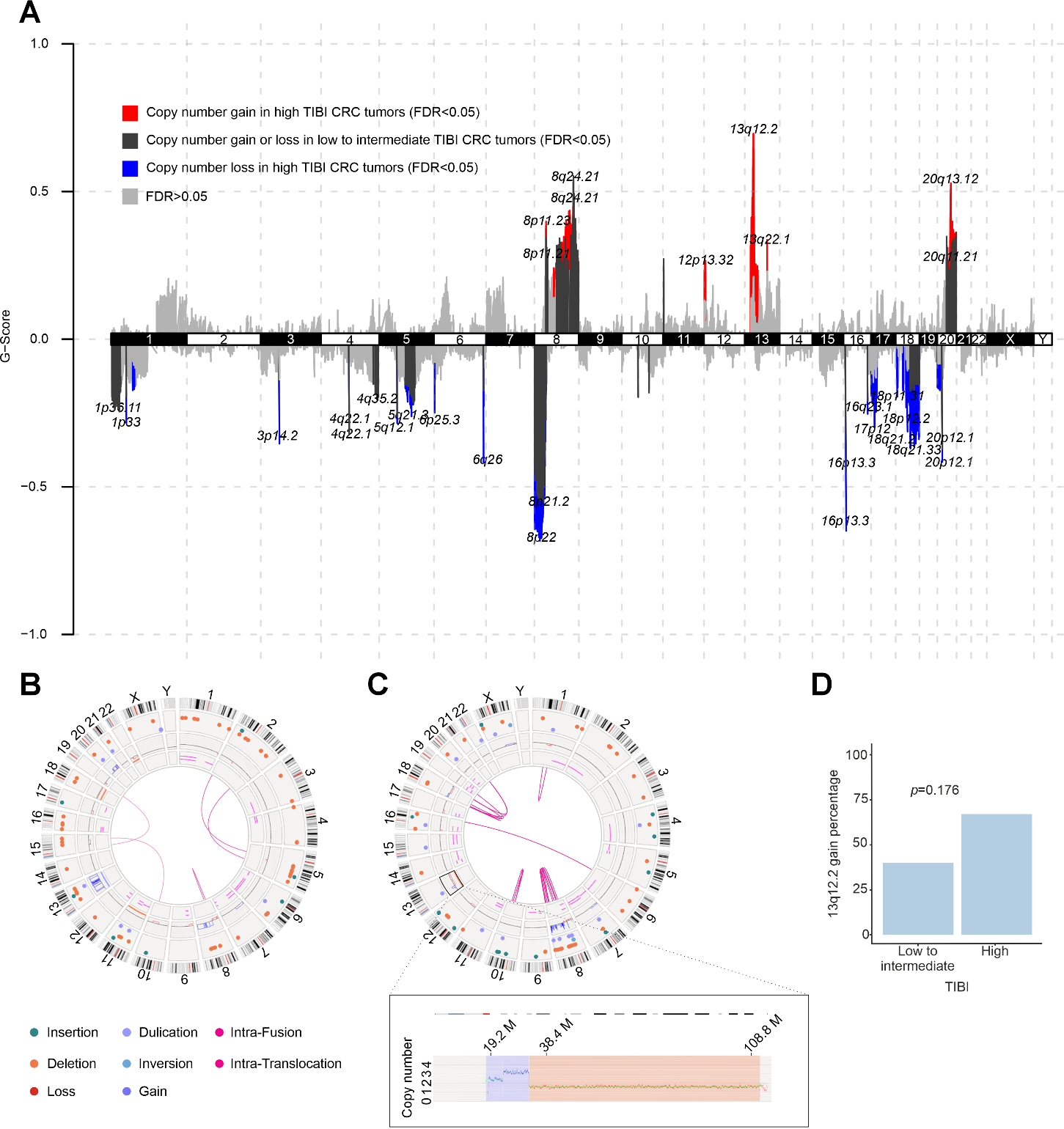


**Figure S7. Chromosome copy-number variation related to infiltrative growth pattern.** (A) GISTIC analysis in The Cancer Genome Atlas (TCGA) colorectal cancers (CRCs) (*n* = 189). (B–D) Optical genomic mapping for the analysis of detailed chromosome structural alterations in 35 patients from cohort 2. (B,C) Circos plots showing structural variation in two representative cases: case (B) shows a gain of the entire chromosome 13q, while (C) shows a more localized gain encompassing chromosome 13q12.2. (D) Bar plots comparing the proportion of cases with 13q12.2 gain across Tumor Invasive Border Index (TIBI) categories. *P*-values are for Fisher’s test.


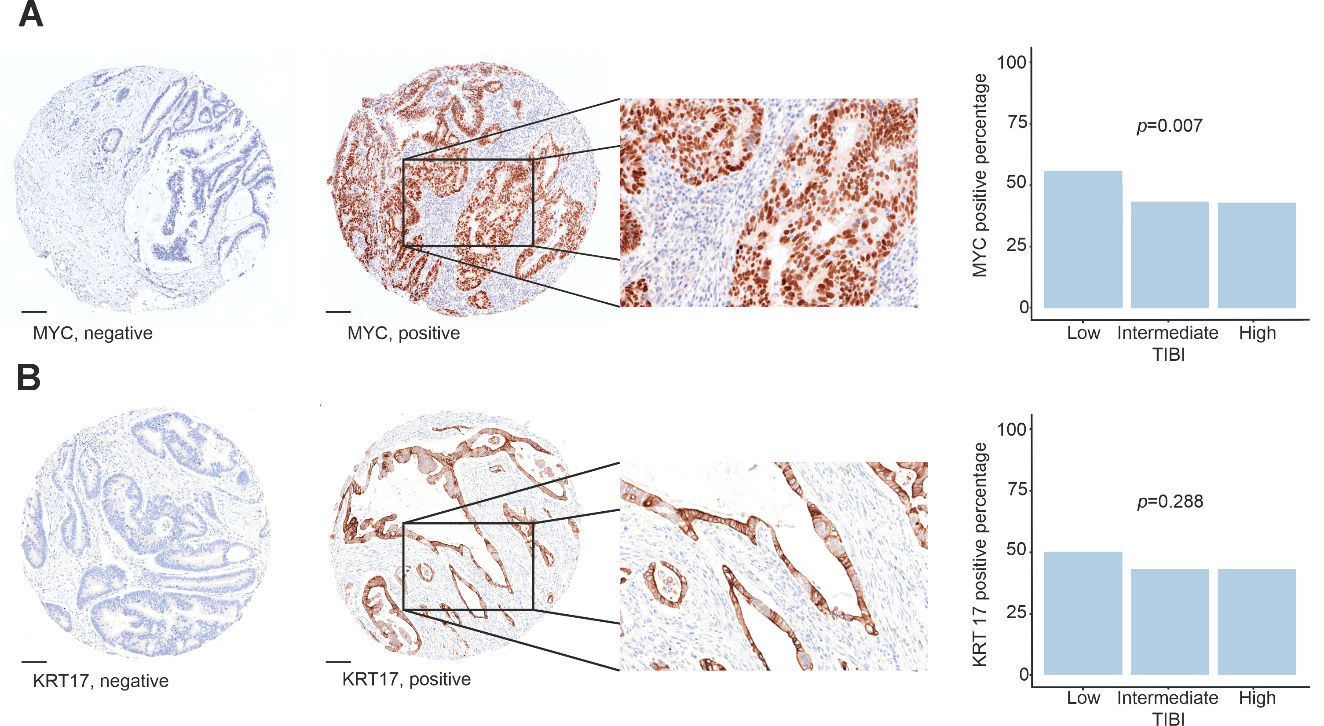


**Figure S8. Associations between Tumor Invasive Border Index (TIBI) and tumor molecular features.** Representative examples of immunohistochemistry staining patterns for (A) MYC (*n* = 760) and (B) KRT17 (*n* = 759), alongside bar charts showing their association with TIBI classification. *P*-values are for Fisher’s test. Scale bars, 100 µm.


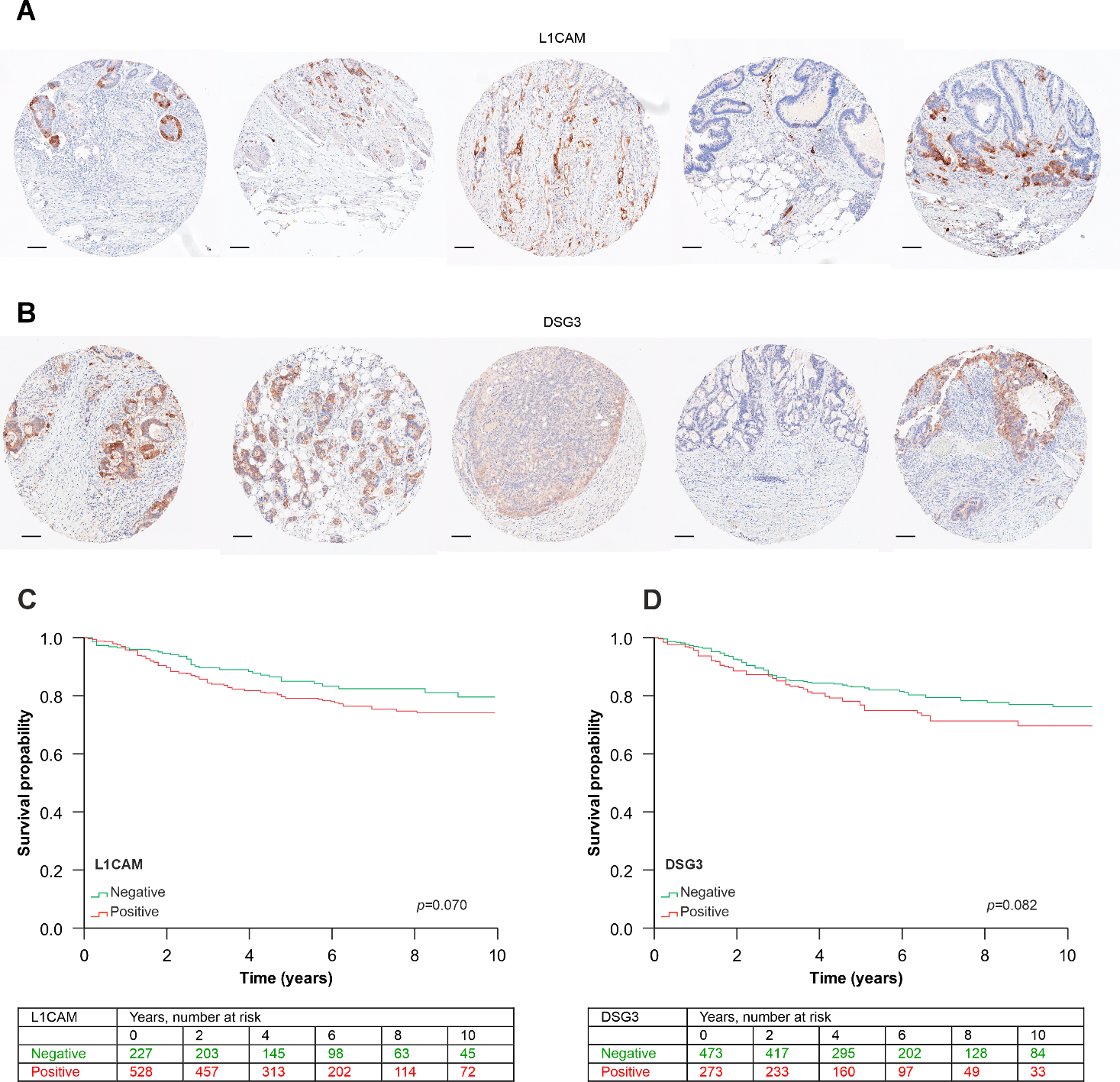


**Figure S9. L1CAM and DSG3 immunohistochemistry at the tumor margin.** Representative examples of (A) L1CAM and (B) DSG3 immunohistochemistry at the tumor invasive border. (C,D) Kaplan–Meier curves on cancer-specific survival based on (C) L1CAM and (D) DSG3 expression. Scale bars, 100 µm.


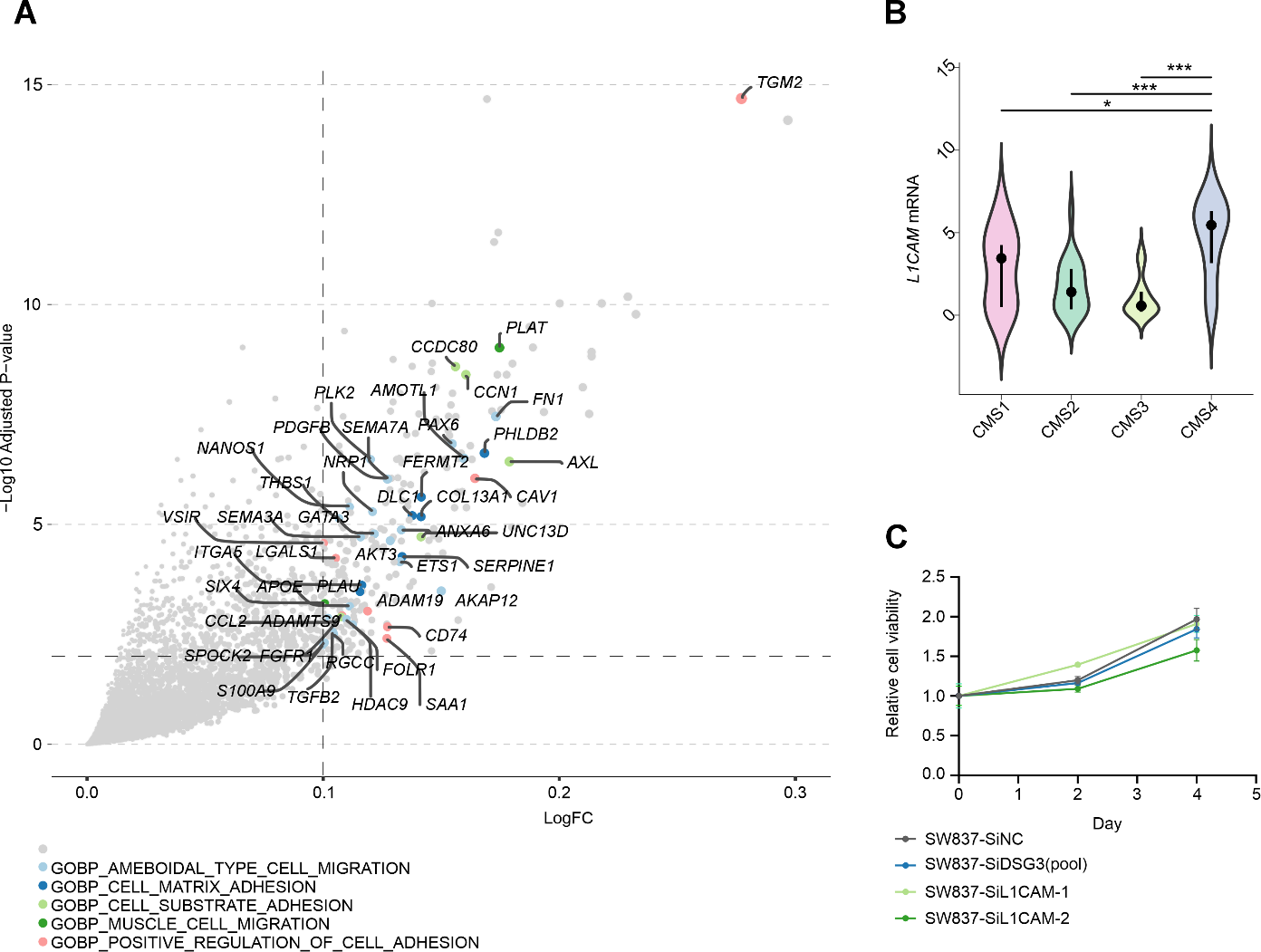


**Figure S10. Additional data from the Cancer Cell Line Encyclopedia (CCLE) dataset and cell culture experiments**. (A) Volcano plot showing genes associated with *L1CAM* expression in the CCLE dataset (*n* = 84 cell lines). (B) Violin plots comparing *L1CAM* expression across Consensus Molecular Subtype (CMS) categories (*n* = 84 cell lines). (C) Scatterplot demonstrating a correlation between *L1CAM* and *ITGA5* expression. (D) Relative cell viability following siRNA treatment. Data are derived from three independent experiments, normalized to day 0 for each group, and presented as mean ± SEM in the graph. **p* < 0.05; ***p* < 0.01; ****p* < 0.001.


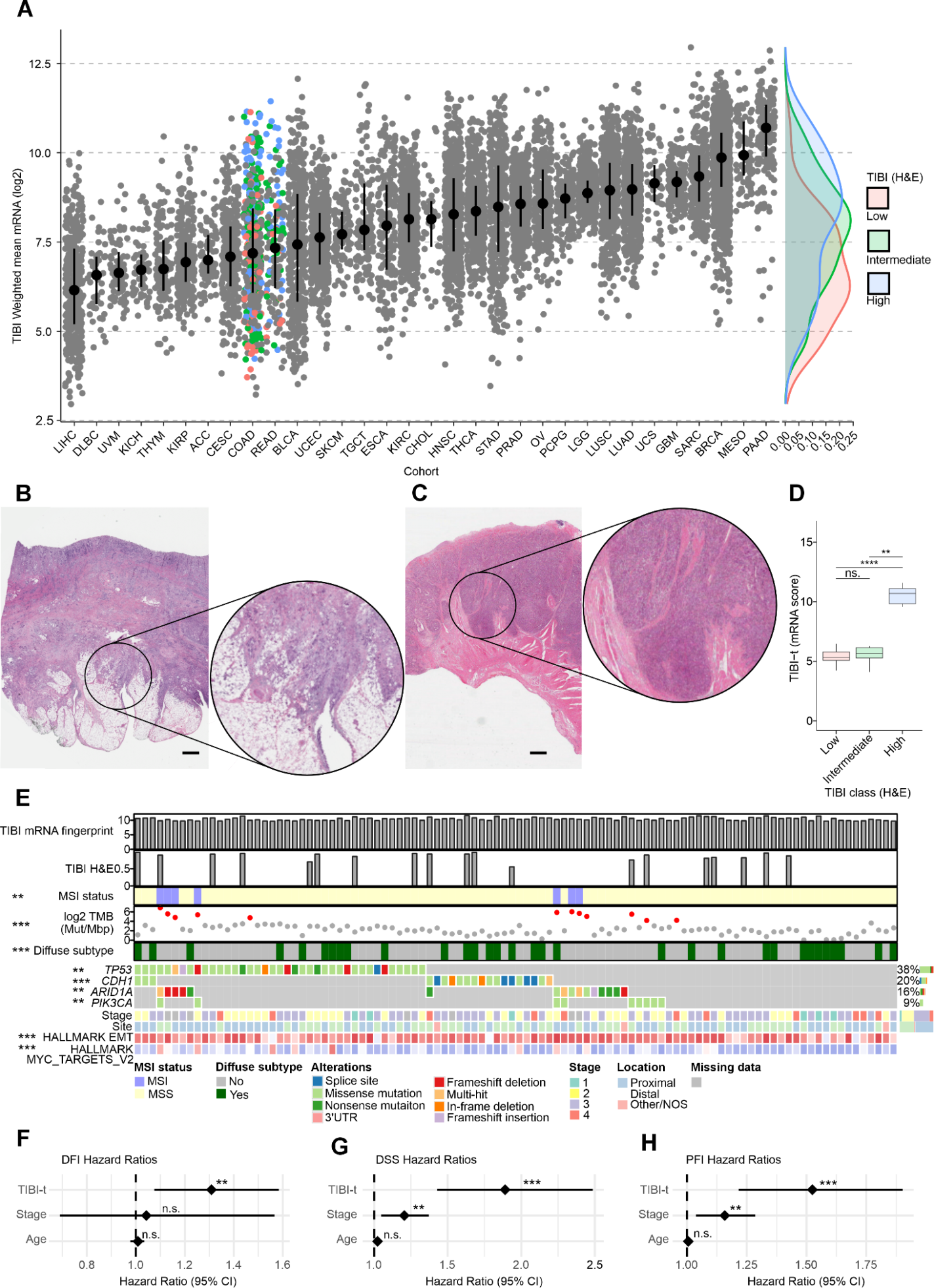


**Figure S11. Pan-cancer and gastric cancer analysis of transcriptomic Tumor Invasive Border Index (TIBI-t) signature. (**A) Pan-cancer analysis of TIBI-t across multiple TCGA cohorts. (B,C) Two representative H&E-stained sections of The Cancer Genome Atlas (TCGA) STAD (B, infiltrative and C, expanding). (D) TIBI-t score across TIBI categories defined from H&E-stained sections. (E) Heatmap demonstrating the mutational landscape and selected clinical features of high TIBI-t STAD cases. (F–H) Multivariable Cox regression models for (F) disease-free interval, (G) disease-specific survival and (H) progression-free interval. **p* < 0.05, ***p* < 0.01, ****p* < 0.001. *P*-values were based on Mann–Whitney U test (panel D), Fisher’s test (panel E), or Cox regression (panels F–H). Scale bars, 1 mm. TCGA cohort abbreviations in panel (A) are defined in the supplementary material, Table S5.

**Table S1.** Cox regression models for cancer-specific survival according to ITCBB tumor budding classification and tumor border TIBI configuration.

|  |  | **Colorectal cancer-specific survival** | | | |
| --- | --- | --- | --- | --- | --- |
|  | No of cases | No of events | Model 1 (univariable)  HR (95% CI) | Model 2 (multivariable)  HR (95% CI) | Model 3 (multivariable)  HR (95% CI) |
| **Cohort 1** |  |  |  |  |  |
| TIBI |  |  |  |  |  |
| Low (< 40%) | 333 | 54 | 1 (reference) | 1 (reference) | 1 (reference) |
| Intermediate (40%–67%) | 373 | 85 | 1.43 (1.02–2.01) | 1.29 (0.92–1.82) | 0.99 (0.69–1.43) |
| High (> 67%) | 357 | 157 | 3.47 (2.54–4.72) | 2.66 (1.92–3.70) | 1.43 (1.00–2.06) |
| *P_trend_* |  |  | < 0.001 | < 0.001 | 0.017 |
| ITBCC tumor budding |  |  |  |  |  |
| Low | 803 | 175 | 1 (reference) | 1 (reference) | 1 (reference) |
| Intermediate | 148 | 61 | 2.24 (1.68–3.00) | 1.72 (1.27–2.32) | 1.21 (0.88–1.65) |
| High | 112 | 60 | 3.20 (2.39–4.30) | 2.22 (1.62–3.03) | 1.24 (0.90–1.71) |
| *P_trend_* |  |  | < 0.001 | < 0.001 | 0.15 |
| **Cohort 2** |  |  |  |  |  |
| TIBI |  |  |  |  |  |
| Low (< 40%) | 218 | 20 | 1 (reference) | 1 (reference) | 1 (reference) |
| Intermediate (40%–67%) | 301 | 50 | 1.91 (1.09–3.33) | 1.69 (0.96–2.96) | 1.73 (0.95–3.14) |
| High (> 67%) | 252 | 76 | 4.70 (2.79–7.91) | 3.41 (1.98–5.88) | 2.03 (1.11–3.71) |
| *P_trend_* |  |  | < 0.001 | < 0.001 | 0.029 |
| ITBCC tumor budding |  |  |  |  |  |
| Low | 538 | 97 | 1 (reference) | 1 (reference) | 1 (reference) |
| Intermediate | 127 | 11 | 2.13 (1.40–3.23) | 1.57 (1.02–2.42) | 1.38 (0.87–2.19) |
| High | 106 | 10 | 3.97 (2.72–5.79) | 2.81 (1.89–4.17) | 1.67 (1.08–2.58) |
| *P_trend_* |  |  | < 0.001 | < 0.001 | 0.019 |

Model 1 was a univariable Cox proportional hazard regression model, model 2 included TIBI and ITBCC, and model 3 was based on model 2 and additionally adjusted for sex, age (< 65, 65–75, > 75), year of operation (2000–2005, 2006–2010, 2011–2015, 2016–2020), tumor location (proximal colon, distal colon, rectum), AJCC T class (T1–2, T3–4), AJCC N class (N0, N1–2) AJCC M class (M0, M1), tumor grade (well/moderately differentiated, poorly differentiated), lymphovascular invasion (negative, positive), mismatch repair (MMR) status (proficient, deficient), *BRAF* status (wild-type, mutant). Missing data for *BRAF* status (*n* = 1 in cohort 1, *n* = 7 in cohort 2) were included in the majority category (*BRAF* wild-type).

*P*_trend_ values were calculated by using the three ordinal categories of TIBI as continuous variables in univariable and multivariable Cox proportional hazard regression models.

AJCC, American Joint Committee on Cancer; ITBCC, International Tumor Budding Consensus Conference; TIBI, Tumor Invasive Border Index.

**Table S2.** Multivariable Cox regression models of cancer-specific survival and overall survival according to TIBI levels and other covariates.

|  | **Cohort 1** | | |  | **Cohort 2** | | |
| --- | --- | --- | --- | --- | --- | --- | --- |
|  | Cancer-specific survival |  | Overall survival |  | Cancer-specific survival |  | Overall survival |
|  | Multivariable  HR (95% CI) |  | Multivariable  HR (95% CI) |  | Multivariable  HR (95% CI) |  | Multivariable  HR (95% CI) |
| TIBI |  |  |  |  |  |  |  |
| Low (< 40%) | 1 (reference) |  | 1 (reference) |  | 1 (reference) |  | 1 (reference) |
| Intermediate (40%–67%) | 0.99 (0.69–1.43) |  | 0.89 (0.70–1.13) |  | 1.73 (0.95–3.14) |  | 1.16 (0.82–1.64) |
| High (> 67%) | 1.43 (1.00–2.06) |  | 1.25 (0.97–1.60) |  | 2.03 (1.11–3.71) |  | 1.20 (0.83–1.74) |
| Age (years) |  |  |  |  |  |  |  |
| < 65 | 1 (reference) |  | 1 (reference) |  | 1 (reference) |  | 1 (reference) |
| 65–75 | 1.18 (0.88–1.59) |  | 1.38 (1.08–1.77) |  | 1.69 (1.10–2.58) |  | 1.76 (1.22–2.53) |
| > 75 | 1.94 (1.44–2.60) |  | 3.09 (2.45–3.90) |  | 2.84 (1.84–4.39) |  | 4.47 (3.17–6.31) |
| Gender |  |  |  |  |  |  |  |
| Male | 1 (reference) |  | 1 (reference) |  | 1 (reference) |  | 1 (reference) |
| Female | 0.92 (0.72–1.17) |  | 0.77 (0.65–0.93) |  | 0.97 (0.69–1.36) |  | 0.82 (0.64–1.05) |
| Year of operation |  |  |  |  |  |  |  |
| 2000–2005 | 1 (reference) |  | 1 (reference) |  | - |  | - |
| 2006–2010 | 0.58 (0.44 –0.77) |  | 0.67 (0.54–0.83) |  | 1 (reference) |  | 1 (reference) |
| 2011–2015 | 0.44 (0.33–0.59) |  | 0.58 (0.47–0.72) |  | 0.93 (0.61–1.41) |  | 0.92 (0.68–1.25) |
| 2016–2020 | - |  | - |  | 0.56 (0.36–0.87) |  | 0.65 (0.46–0.90) |
| Tumor location |  |  |  |  |  |  |  |
| Proximal colon | 1 (reference) |  | 1 (reference) |  | 1 (reference) |  | 1 (reference) |
| Distal colon | 0.94 (0.72–1.22) |  | 0.99 (0.81–1.21) |  | 1.17 (0.77–1.80) |  | 1.06 (0.76–1.47) |
| Rectum | 0.91 (0.63–1.31) |  | 0.95 (0.72–1.26) |  | 0.86 (0.55–1.33) |  | 0.97 (0.70–1.34) |
| AJCC T class |  |  |  |  |  |  |  |
| T1–2 | 1 (reference) |  | 1 (reference) |  | 1 (reference) |  | 1 (reference) |
| T3–4 | 2.30 (1.40–3.78) |  | 1.31 (1.01–1.70) |  | 1.03 (0.58–1.82) |  | 0.98 (0.71–1.37) |
| AJCC N class |  |  |  |  |  |  |  |
| N0 | 1 (reference) |  | 1 (reference) |  | 1 (reference) |  | 1 (reference) |
| N1–N2 | 2.21 (1.66–2.95) |  | 1.38 (1.31–1.69) |  | 1.94 (1.24–3.05) |  | 1.19 (0.89–1.61) |
| AJCC M class |  |  |  |  |  |  |  |
| M0 | 1 (reference) |  | 1 (reference) |  | 1 (reference) |  | 1 (reference) |
| M1 | 6.53 (4.96–8.59) |  | 5.40 (4.28–6.81) |  | 8.73 (5.90–12.91) |  | 6.60 (4.74–9.20) |
| Tumor grade |  |  |  |  |  |  |  |
| Low-grade | 1 (reference) |  | 1 (reference) |  | 1 (reference) |  | 1 (reference) |
| High-grade | 1.85 (1.37–2.49) |  | 1.90 (1.51–2.40) |  | 1.36 (0.89–2.10) |  | 1.09 (0.78–1.55) |
| Lymphovascular invasion |  |  |  |  |  |  |  |
| No | 1 (reference) |  | 1 (reference) |  | 1 (reference) |  | 1 (reference) |
| Yes | 1.58 (1.22–2.05) |  | 1.38 (1.12–1.71) |  | 1.85 (1.16–2.95) |  | 1.34 (0.99–1.81) |
| MMR status |  |  |  |  |  |  |  |
| MMR proficient | 1 (reference) |  | 1 (reference) |  | 1 (reference) |  | 1 (reference) |
| MMR deficient | 0.67 (0.40–1.10) |  | 0.75 (0.53–1.06) |  | 0.56 (0.27–1.17) |  | 0.98 (0.63–1.52) |
| *BRAF* status |  |  |  |  |  |  |  |
| Wild-type | 1 (reference) |  | 1 (reference) |  | 1 (reference) |  | 1 (reference) |
| Mutant | 1.25 (0.88–1.65) |  | 1.38 (1.01–1.87) |  | 1.70 (0.94–3.09) |  | 1.47 (0.97–2.25) |
| Tumor budding (ITBCC) |  |  |  |  |  |  |  |
| Low | 1 (reference) |  | 1 (reference) |  | 1 (reference) |  | 1 (reference) |
| Intermediate | 1.21 (0.88–1.65) |  | 1.27 (1.00–1.62) |  | 1.38 (0.87–2.19) |  | 1.25 (0.86–1.80) |
| High | 1.24 (0.90–1.71) |  | 1.19 (0.91–1.56) |  | 1.67 (1.08–2.58) |  | 1.54 (1.09–2.19) |

Missing data for *BRAF* status (*n* = 1 in cohort 1, *n =*7 in cohort 2) were included in the majority category (wild-type) to limit the degrees of freedom.

AJCC, American Joint Committee on Cancer; MMR, mismatch repair, ITBCC; Internation Tumor Budding Consensus Conference; TIBI, Tumor Invasive Border Index.

**Table S3.** Univariable and multivariable Cox regression models for cancer-specific and overall survival according to L1CAM and DSG3 expression in cohort 2.

|  |  | **Colorectal cancer-specific survival** | | | | **Overall survival** | | |
| --- | --- | --- | --- | --- | --- | --- | --- | --- |
|  | No of cases | No of events | Univariable  HR (95% CI) | Multivariable  HR (95 % CI) |  | No of events | Univariable  HR (95% CI) | Multivariable  HR (95% CI) |
| **L1CAM** |  |  |  |  |  |  |  |  |
| Negative | 333 | 54 | 1 (reference) | 1 (reference) |  | 147 | 1 (reference) | 1 (reference) |
| Positive | 373 | 85 | 1.42 (0.97–2.09) | 1.16 (0.76–1.76) |  |  | 1.06 (0.81–1.38) | 0.97 (0.72–1.30) |
| *p_trend_* |  |  | 0.072 | 0.50 |  |  | 0.67 | 0.85 |
| **DSG3** |  |  |  |  |  |  |  |  |
| Negative | 218 | 20 | 1 (reference) | 1 (reference) |  | 58 | 1 (reference) | 1 (reference) |
| Positive | 301 | 50 | 1.34 (0.96–1.87) | 1.20 (0.84–1.69) |  |  | 1.08 (0.84–1.39) | 1.08 (0.83–1.41) |
| *p_trend_* |  |  | 0.083 | 0.31 |  |  | 0.55 | 0.56 |

Multivariable Cox proportional hazards regression models were adjusted for sex, age (< 65, 65–75, > 75), year of operation (2000–2005, 2006–2010, 2011–2015, 2016–2020), tumor location (proximal colon, distal colon, rectum), AJCC T class (T1-2, T3-4), AJCC N class (N0, N1-2) AJCC M class (M0, M1), tumor grade (low-grade, high-grade), lymphovascular invasion (negative, positive), mismatch repair (MMR) status (proficient, deficient), *BRAF* status (wild-type, mutant). Missing data on *BRAF* status (*n* = 1 in cohort 1, *n* = 7 in cohort 2) were included in the major category (*BRAF* wild-type) to reduce the degrees of freedom.

*p_trend_* values were calculated by using the two ordinal categories of DSG3 and L1CAM as continuous variables in univariable and multivariable Cox proportional hazard regression models.

AJCC, American Joint Committee on Cancer; TIBI, Tumor Invasive Border Index.

**Table S4.** Genes chosen for TIBI-t score and their weight in the score.

| **Gene symbol** | **Weight** | **Gene symbol** | **Weight** |
| --- | --- | --- | --- |
| *NPR3* | 1.72071 | *SULF1* | 1.11600 |
| *PGR* | 1.21865 | *ALPK3* | 1.03893 |
| *HTRA3* | 1.13450 | *ITGA11* | 1.02761 |
| *GSTM5* | 1.03801 | *CCDC80* | 1.09762 |
| *FAM19A5* | 1.04466 | *COL1A1* | 1.01860 |
| *CCDC8* | 1.06295 | *CLDN11* | 1.00173 |
| *MMP11* | 1.23009 | *PRELP* | 1.36927 |
| *AEBP1* | 1.19214 | *CDO1* | 1.01165 |
| *BGN* | 1.09351 | *FGF14* | 1.12993 |
| *AHNAK2* | 1.21875 | *CDH2* | 1.18924 |
| *ODZ4* | 1.18144 | *GAS1* | 1.27635 |
| *PEG3* | 1.06885 | *COL8A1* | 1.19426 |
| *LTBP2* | 1.02606 | *SOX10* | 1.09674 |
| *KIF26B* | 1.24153 | *RSPO3* | 1.12337 |
| *P4HA3* | 1.22702 | *PTPRN* | 1.07420 |
| *ISLR* | 1.16875 | *BNC2* | 1.00213 |
| *DPYSL4* | 1.06979 | *COL11A1* | 1.42345 |
| *ANTXR1* | 1.10993 | *ASPN* | 1.22825 |
| *SPTBN5* | 1.13322 | *PHACTR3* | 1.30269 |
| *HMCN1* | 1.21661 | *ITGBL1* | 1.36966 |
| *GLI3* | 1.10062 | *MATN3* | 1.19779 |
| *COL1A2* | 1.04365 | *CNTN1* | 1.09722 |
| *L1CAM* | 1.61803 | *OMD* | 1.24328 |
| *ZFPM2* | 1.07331 | *CTHRC1* | 1.00895 |
| *SPOCK1* | 1.46418 | *DCHS2* | 1.16110 |
| *ACSS3* | 1.15728 | *DDR2* | 1.05334 |
| *HECW1* | 1.05734 | *COMP* | 1.67278 |
| *MGP* | 1.15255 | *NGFR* | 1.02989 |
| *COL10A1* | 1.89111 | *LONRF2* | 1.10870 |
| *LRRC4C* | 1.02731 | *LGR6* | 1.10251 |
| *CSDC2* | 1.29314 | *PLXNA4* | 1.02301 |
| *THBS2* | 1.39207 | *UCHL1* | 1.02015 |
| *SSC5D* | 1.16458 | *ADAMTS16* | 1.08570 |
| *NCAM2* | 1.18808 | *IFI44L* | 1.03273 |
| *FNDC1* | 1.38838 | *COLEC12* | 1.03689 |
| *GFPT2* | 1.28044 | *C3* | 1.00943 |
| *SCN2B* | 1.02536 | *ZFHX4* | 1.02077 |
| *SFRP4* | 1.70537 | *EPHB1* | 1.02268 |
| *ANK2* | 1.10755 | *PDZRN4* | 1.00007 |
| *NTRK2* | 1.51803 | *HSPB7* | 1.10150 |
| *PRG4* | 1.13135 | *POSTN* | 1.00059 |
| *PRRX1* | 1.01885 |  |  |

TIBI-t, transcriptomic Tumor Invasive Border Index.

**Table S5**. TCGA cohort abbreviations shown in the supplementary material, Figure S11.

| **Abbreviation** | **Full cohort name** |
| --- | --- |
| LIHC | Liver Hepatocellular Carcinoma |
| DLBC | Lymphoid Neoplasm Diffuse Large B-cell Lymphoma |
| UVM | Uveal Melanoma |
| KICH | Kidney Chromophobe |
| THYM | Thymoma |
| KIRP | Kidney Renal Papillary Cell Carcinoma |
| ACC | Adrenocortical Carcinoma |
| CESC | Cervical Squamous Cell Carcinoma and Endocervical Adenocarcinoma |
| COAD | Colon Adenocarcinoma |
| READ | Rectum Adenocarcinoma |
| BLCA | Bladder Urothelial Carcinoma |
| UCEC | Uterine Corpus Endometrial Carcinoma |
| SKCM | Skin Cutaneous Melanoma |
| TGCT | Testicular Germ Cell Tumors |
| ESCA | Esophageal Carcinoma |
| KIRC | Kidney Renal Clear Cell Carcinoma |
| CHOL | Cholangiocarcinoma |
| HNSC | Head and Neck Squamous Cell Carcinoma |
| THCA | Thyroid Carcinoma |
| STAD | Stomach Adenocarcinoma |
| PRAD | Prostate Adenocarcinoma |
| OV | Ovarian Serous Cystadenocarcinoma |
| PCPG | Pheochromocytoma and Paraganglioma |
| LGG | Brain Lower Grade Glioma |
| LUSC | Lung Squamous Cell Carcinoma |
| LUAD | Lung Adenocarcinoma |
| UCS | Uterine Carcinosarcoma |
| GBM | Glioblastoma Multiforme |
| SARC | Sarcoma |
| BRCA | Breast Invasive Carcinoma |
| MESO | Mesothelioma |
| PAAD | Pancreatic Adenocarcinoma |

TCGA, The Cancer Genome Atlas.
