# Supplementary material for: Tumor Invasive Border Index (TIBI) in colorectal cancer: linking infiltrative morphology to molecular insights
Source: J Pathol. 2026 Jun 18;270(1):114–28. doi: 10.1002/path.70087 (PMC13431732; doi:10.1002/path.70087)
Supplement: Supplementary file 3 — File S2. Tumor Invasive Border Index (TIBI) evaluation manual (provided as a separate Word document) [file PATH-270-114-s004.docx]

**Tumor Invasive Border Index (TIBI) in colorectal cancer: linking infiltrative morphology to molecular insights**

A Kehusmaa, J Härkönen, H Li *et al. J Pathol* <https://doi.org/10.1002/path.70087>

**Supplementary File S2.** **Tumor invasive border index (TIBI) evaluation manual**

**TUMOR INVASIVE BORDER INDEX (TIBI) EVALUATION MANUAL**

Contents

[1 Introduction 3](#_Toc221013016)

[2 Definition and criteria for the deepest point of invasive tumor border 4](#_Toc221013017)

[1 Choosing the deepest point of invasion 4](#_Toc221013018)

[2 Special consideration in tumor invasive border configuration: tumor invades the same layer in various places 5](#_Toc221013019)

[3 Special considerations in tumor invasive border configuration: invasive border out of slide 5](#_Toc221013020)

[4 Special considerations in tumor invasive border configuration: mucinous tumors 6](#_Toc221013021)

[3 Tumor Invasive Border Index (TIBI) as a method for evaluating infiltration 8](#_Toc221013022)

[4 Examples 12](#_Toc221013023)

[Example 1 12](#_Toc221013024)

[Example 2 13](#_Toc221013025)

[Example 3 14](#_Toc221013026)

[Example 4 15](#_Toc221013027)

# Introduction

Tumor border configuration (TBC) is a potential prognostic factor in colorectal cancer. TBC can be evaluated using low-power magnification from standard H&E-stained slides of formalin-fixed paraffin-embedded tissue. Infiltrating tumor border has been shown to associate with worse survival compared with an expansile one, but due to the poor reproducibility of the existing criteria, TBC has not frequently been utilized as a prognostic factor in the clinical setting.

**Expansile TBC** is defined as a well circumcised invasive tumor border, that grows bluntly, pushing the tissue on its way, rather than dissecting through the tissue.

**Infiltrating TBC** is defined as an invasive border with no clear boundary between tumor and its surrounding tissue, with tumor dissecting sharply through the tissue surrounding it.


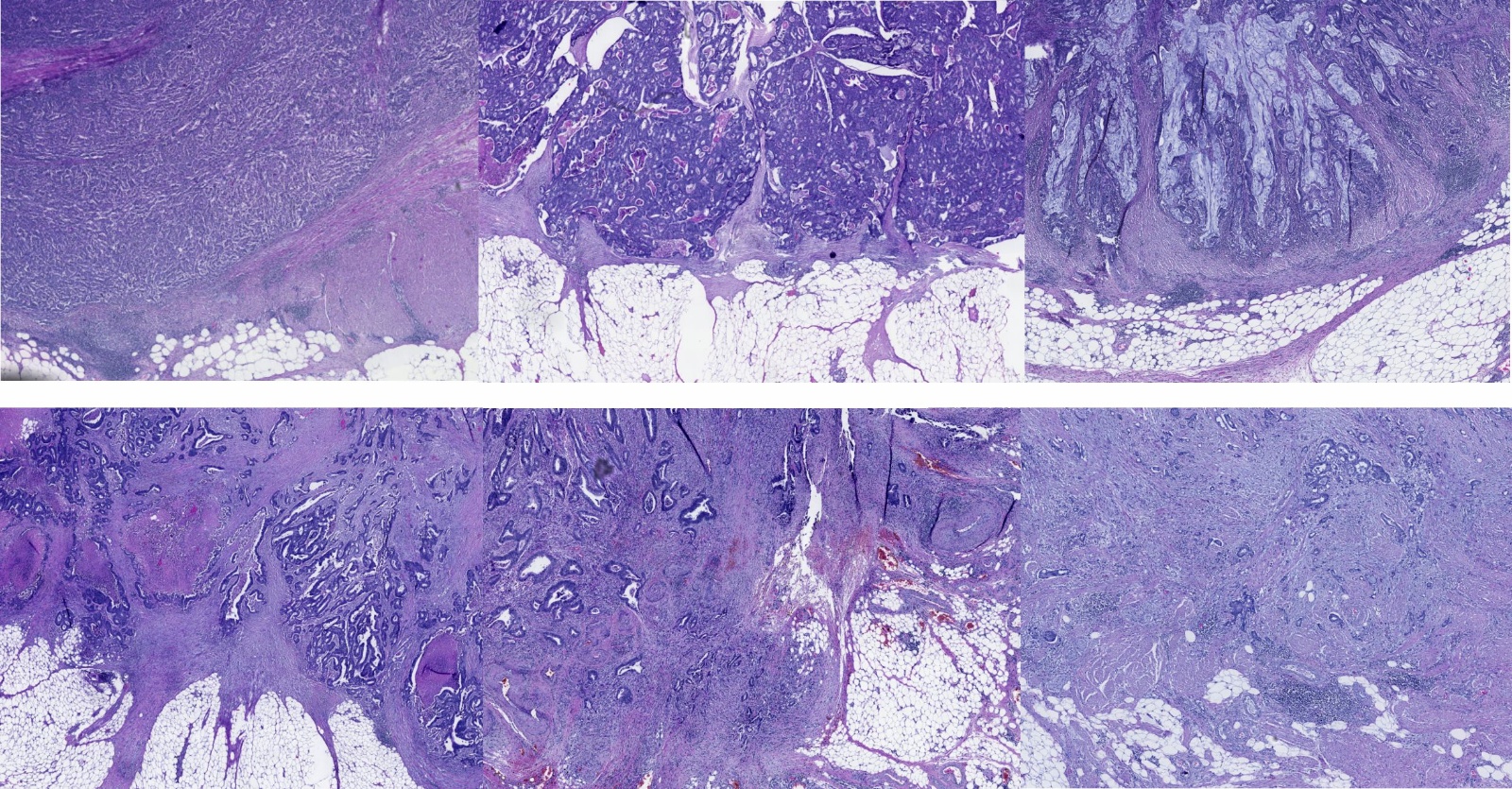


Examples of tumor border configuration ordered from expansile to increasingly infiltrative.

# Definition and criteria for the deepest point of invasive tumor border

## Choosing the deepest point of invasion

TBC assessment is based on visual evaluation of H&E-stained slides. Choose the tumor slide with the deepest invasion denoting the slide that contains malignant cells in the deepest anatomical layer of the intestinal wall. The deepest point of invasion is the area where tumor cells extend farthest beyond the deepest anatomical layer penetrated by the tumor.


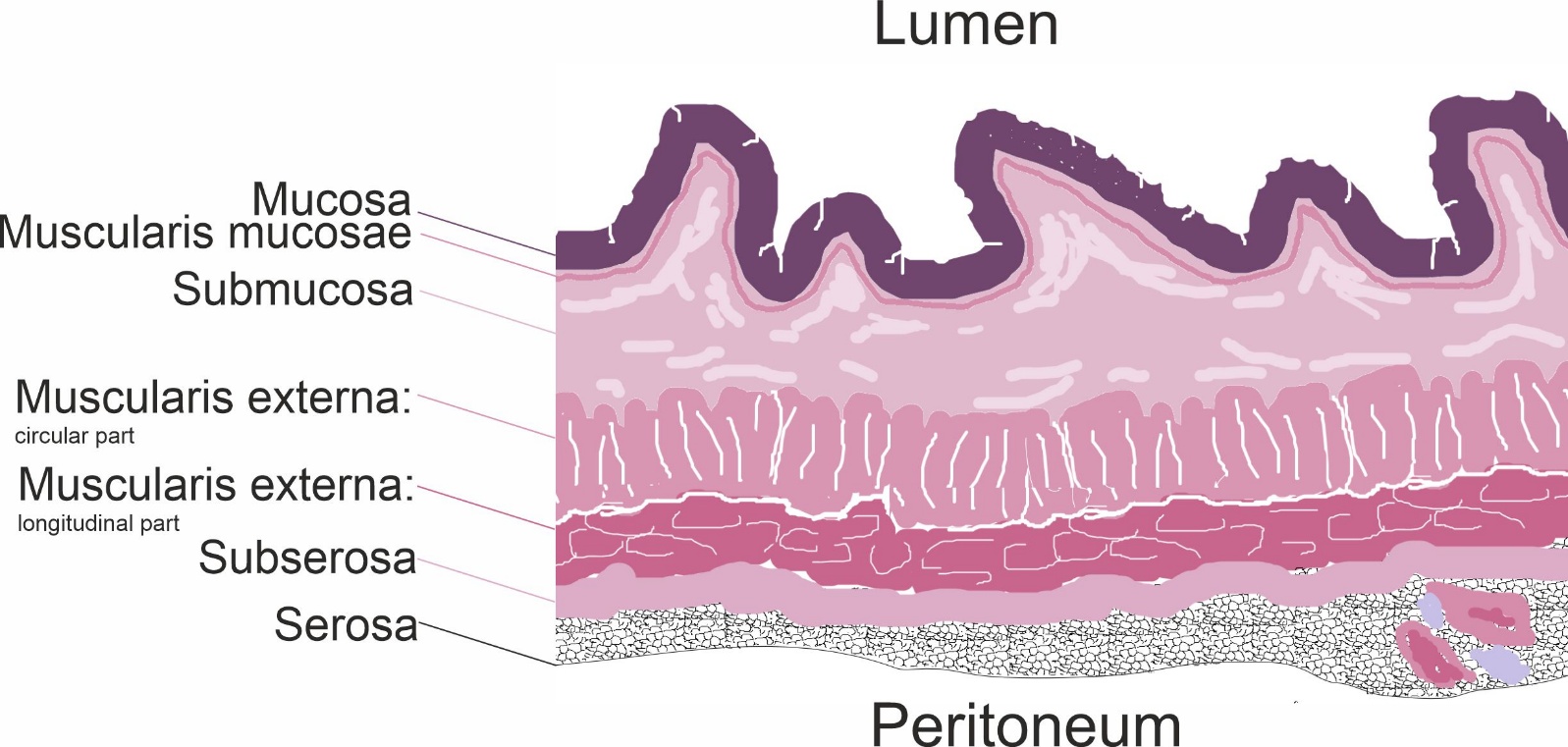


Schematic of the anatomical layers of the colon. The deepest point of invasion is chosen in reference to colon wall anatomical layers.

The following areas should be excluded when choosing the deepest point of invasion:

- Lymph node metastases
- Tumor tissue mislocated at a deeper point as an artefact of tissue sectioning


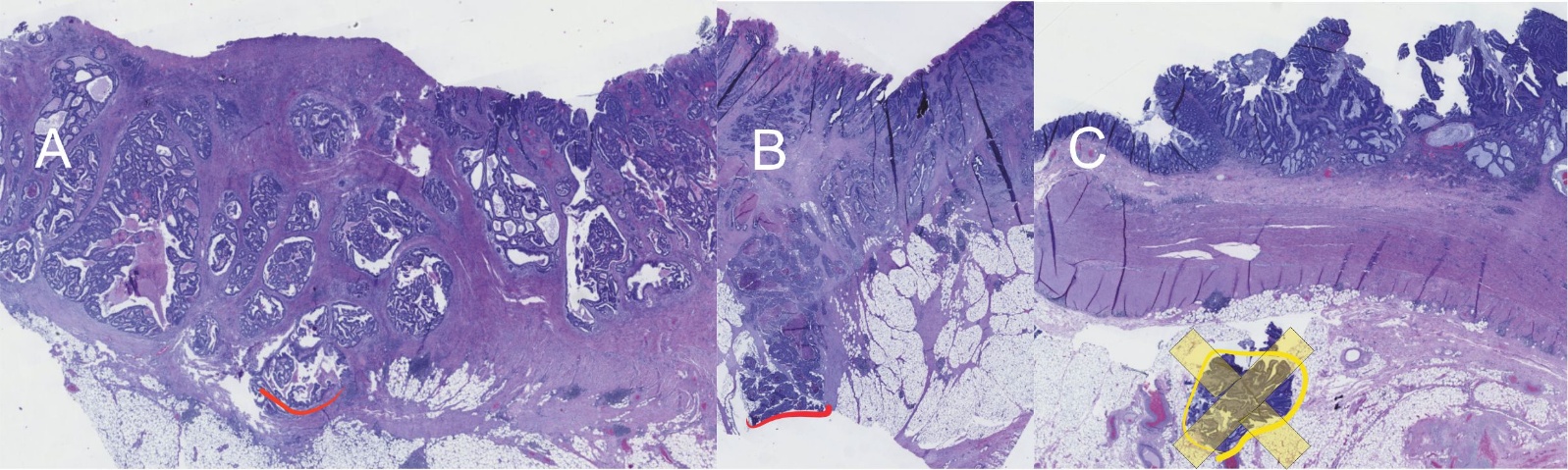


In panel A, the deepest point of invasion, marked in red, represents the area farthest from the muscle layer, which is the deepest layer penetrated by the tumor. In panel B, the region with the deepest point of invasion is the area closest to perforating the intestine wall. The tissue fragment in the yellow circle in panel C should be considered an artefact and not as the deepest point of invasion.

## Special consideration for the configuration of the invasive tumor border: tumor invades the same anatomical layer at multiple sites

Tumors often reach the same anatomical layer in multiple regions simultaneously. If it is unclear which branch of tumor growth extends the farthest beyond the preceding anatomical layer, the area exhibiting the most infiltrative growth should be selected.


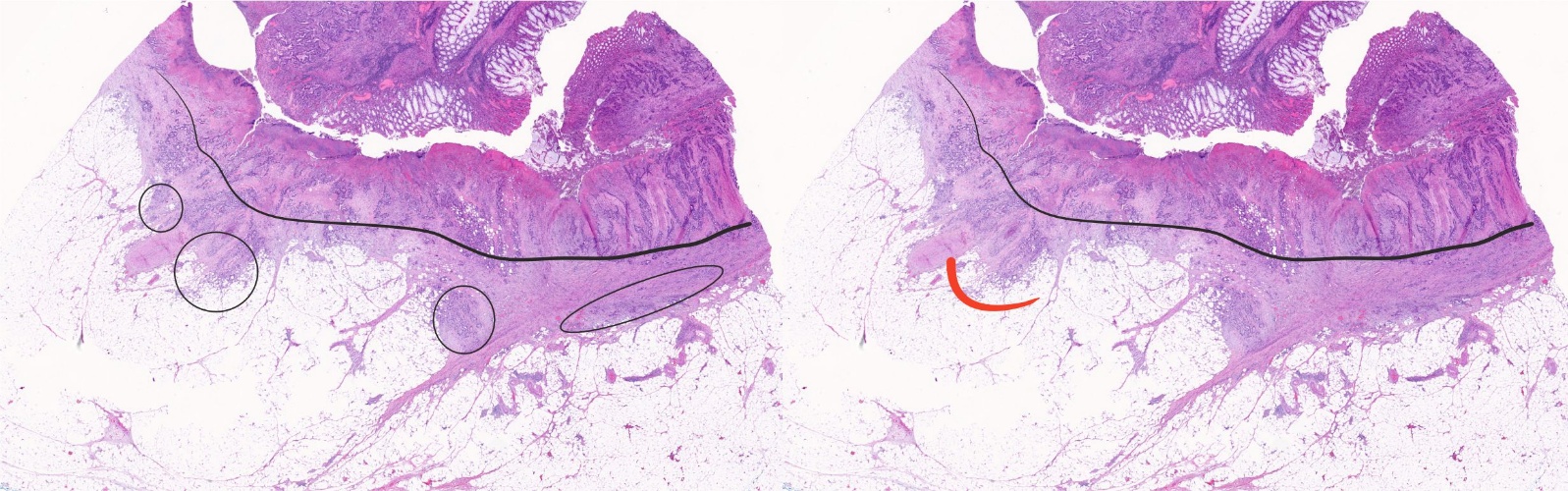


Example of a tumor with several invasive margin foci (black ellipses) at the same depth beyond the deepest penetrated anatomical layer (black line). The red line marks the point that was considered the most infiltrative.

## Special considerations in tumor invasive border configuration: invasive border extending beyond the slide

It can occasionally be difficult to define the deepest point of the invasive border due to the tumor growing outside of the area captured on the H&E slide or invading from multiple different directions. In these cases, the point that best represents the invasive border should be selected near the deepest point of invasion.


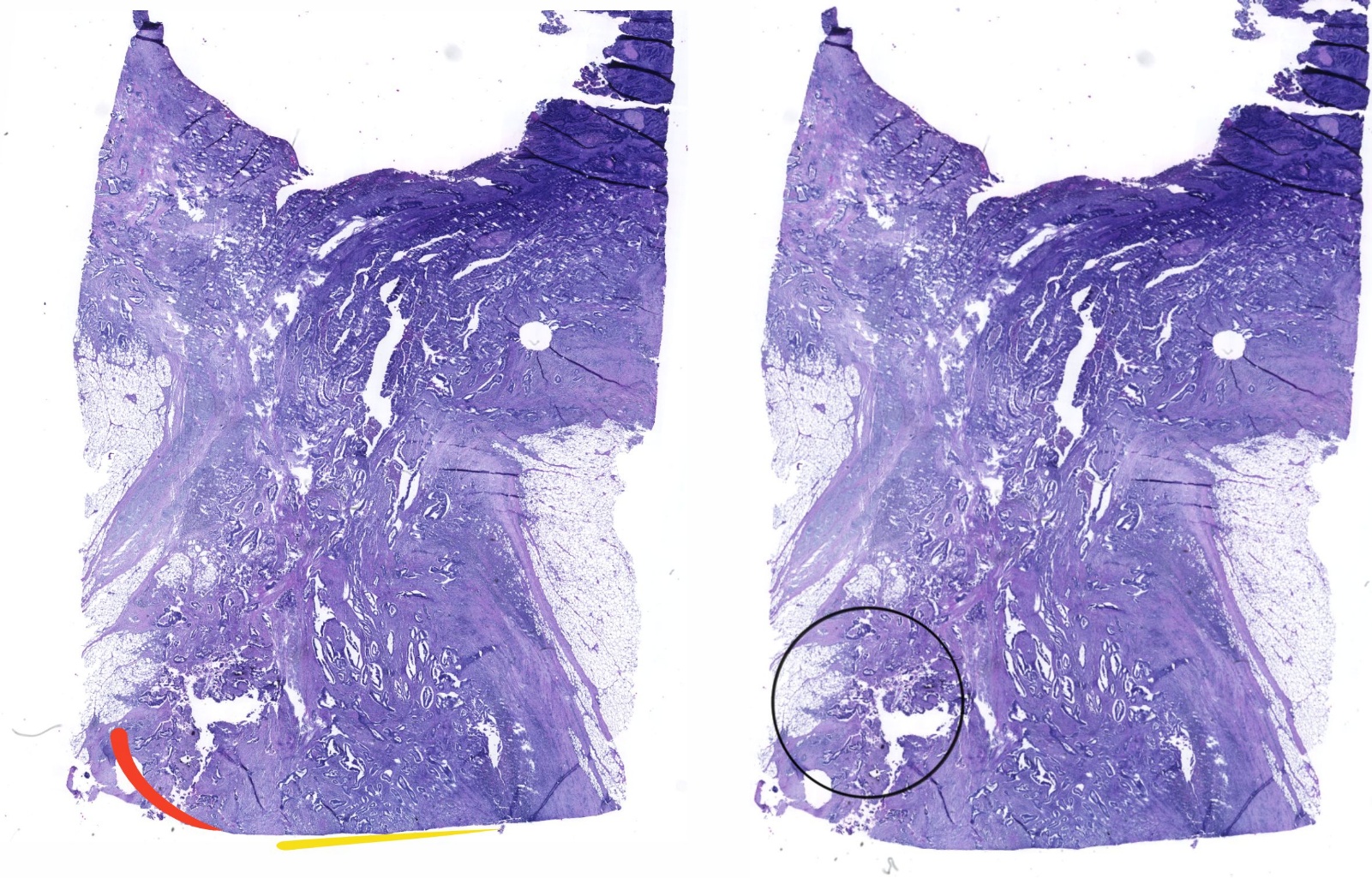


Example of a slide where tumor tissue appears to grow outside of the view, the invasive front marked in yellow above. The region of the invasive front marked red would likely represent the invasive border relatively well.

## Special considerations in tumor invasive border configuration: mucinous tumors

The invasive margin of mucinous tumors or signet ring cell carcinomas can be difficult to evaluate, as the invasive margin may contain pools of acellular mucin. In these cases, the deepest point of invasion should be selected at the border between mucus and the surrounding tissue.


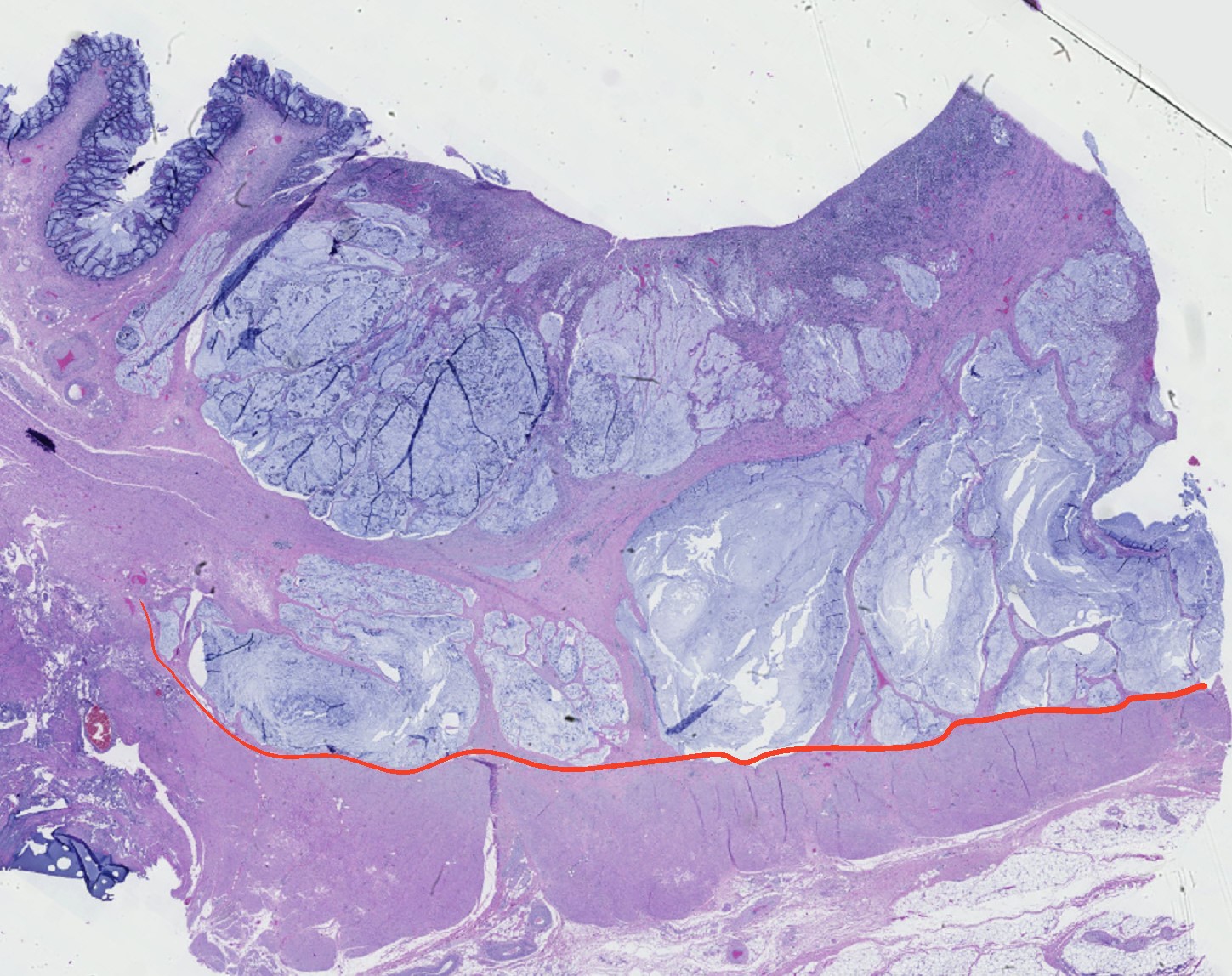


Example of a mucinous tumor, with the invasive border marked by the red line.

# Tumor Invasive Border Index (TIBI) as a method for evaluating infiltration

Tumor Invasive Border Index (TIBI) is a method for evaluating the TBC that is based on relative abundance of different tissue types within a hotspot at the deepest point of invasion.

For the TIBI classification, follow these steps:

(1) Position a 4 mm diameter hotspot, corresponding to a 10× power magnification field of view in a conventional light microscope, at the deepest point of invasion.

- Locate the edge of the hotspot at the deepest tumor cell so that the hotspot covers the tissue in the direction of growth of the tumor, as indicated by the red arrow in the figure below.


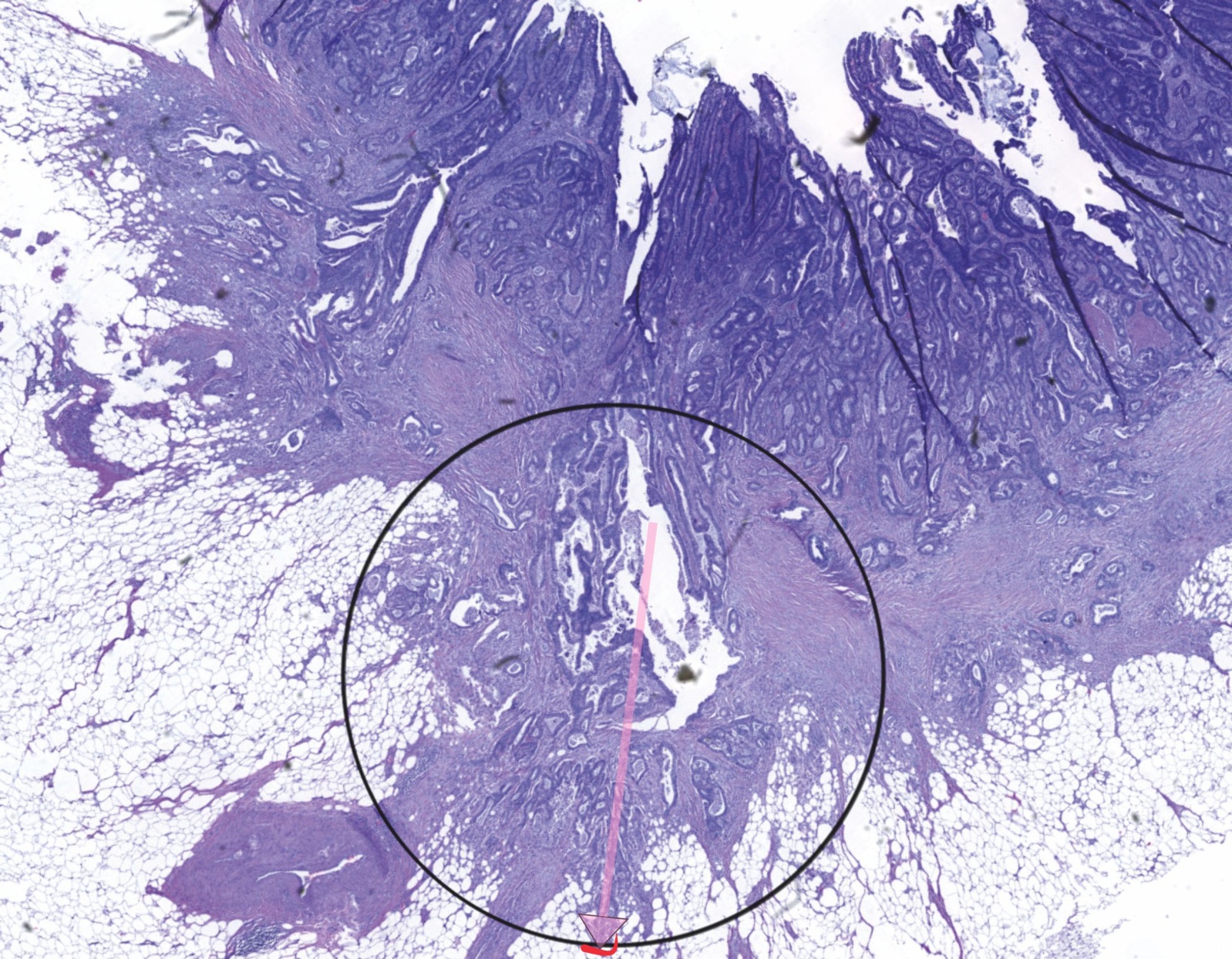


(2) Inside the hotspot, the tissue types to be considered are:

- Tumor epithelium (malignant cells)
- stroma (fibroblasts, infiltrating immune cells, connective tissue)
- mucus
- adipose tissue

Exclude any other tissue types, such as blood vessels, necrosis, muscle, and nerves, as well as all whitespace.


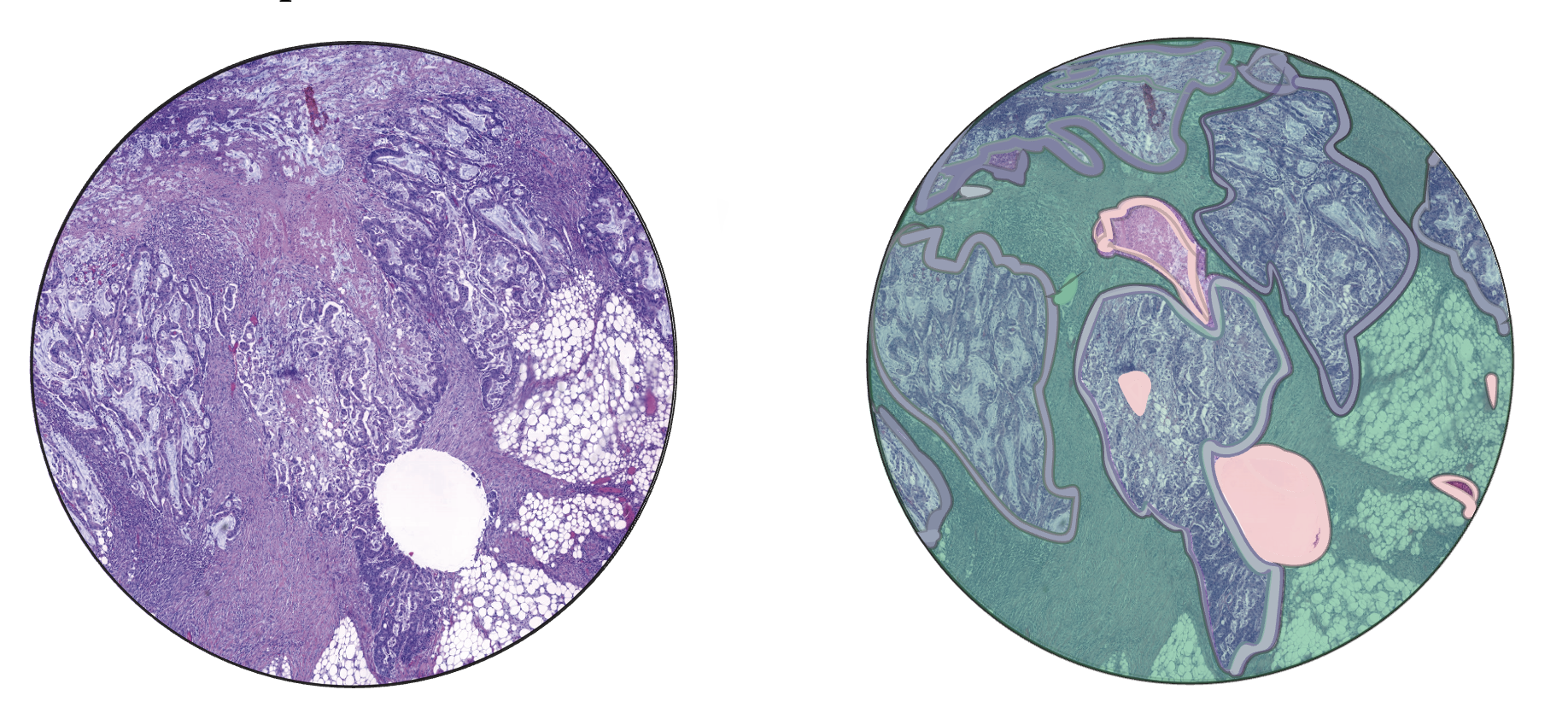
Example of excluding the tissue components irrelevant to TIBI analysis. In the core on the right, excluded tissue types (necrosis, blood vessels) and whitespace are highlighted in light red, whereas included tissue types are marked in shades of green.

(3) Determine TIBI by dividing the total area of stroma and adipose tissue by the total tissue surface area (stroma, adipose tissue, tumor epithelium, mucus), and report the values in five percent increments.

$$TIBI=\frac{A\left( stroma \right)+A(adipose)}{A\left( stroma \right)+A\left( adipose \right)+A\left( tumor \right)+A(mucus)}\times100\%$$

The formula for estimating TIBI.

Then, classify the TIBI as follows:

High: TIBI > 67%

Intermediate: TIBI = 40%–67%

Low: TIBI < 40%

High TIBI is associated with an infiltrative tumor border configuration.

Examples of varying degrees of TIBI in non-mucinous tumors:


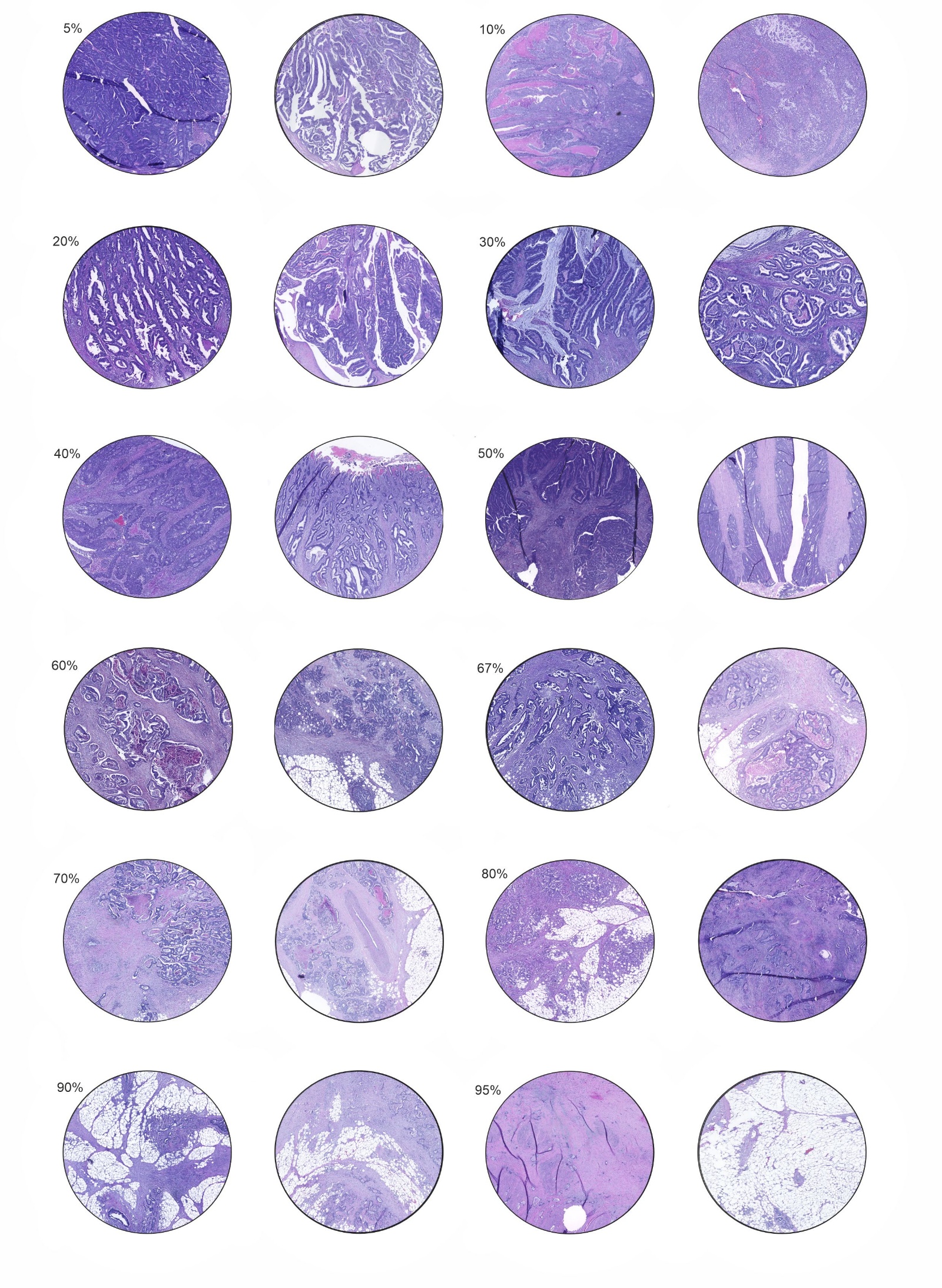


Examples of varying degrees of TIBI in mucinous tumors:


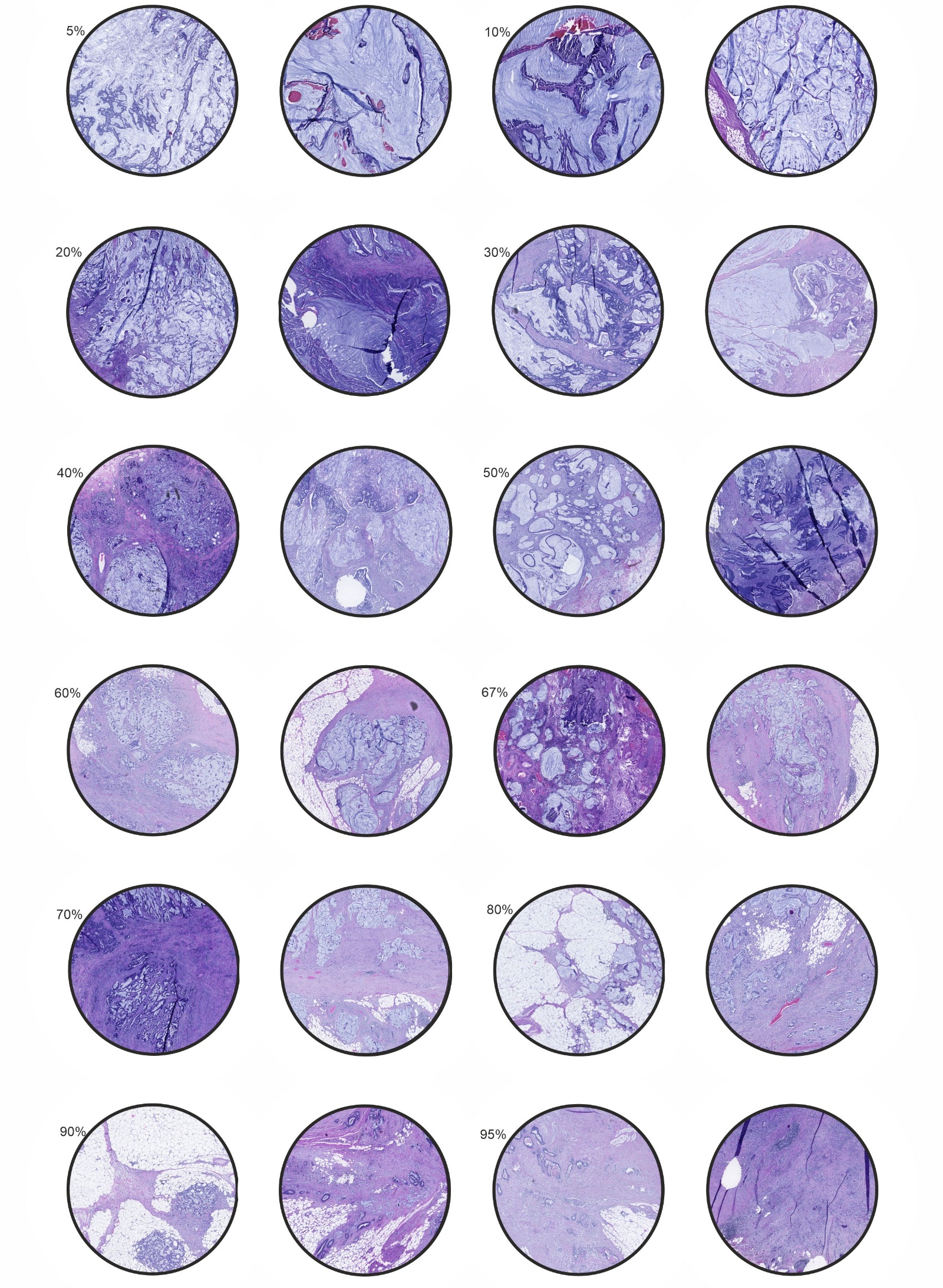


# Examples

## Example 1


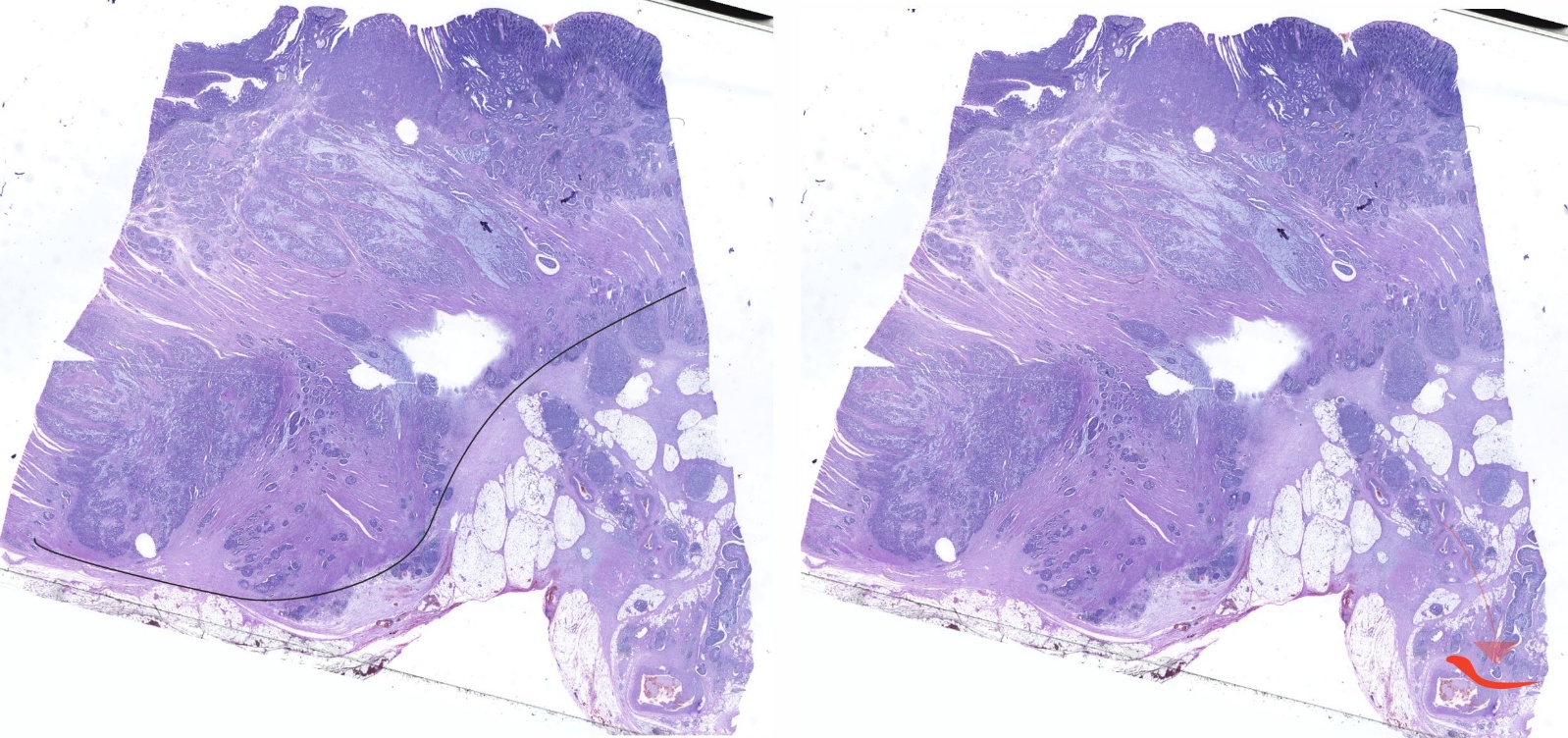


The black line represents the line of the deepest anatomical layer that the cancer has completely passed. The deepest point of invasion is marked with a red line.


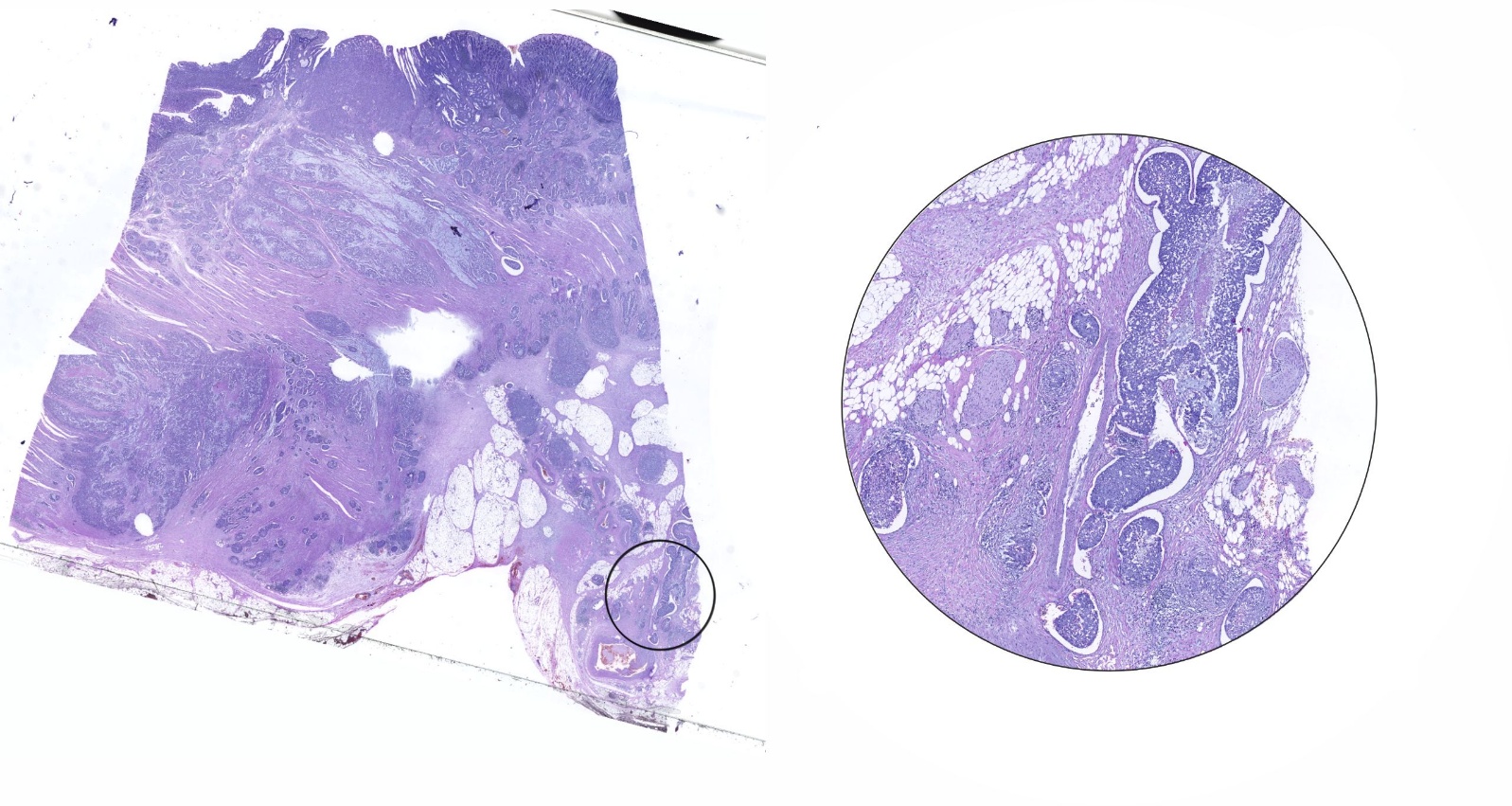


TIBI is estimated to be around 70%, placing it in the ‘high’ category.

## Example 2


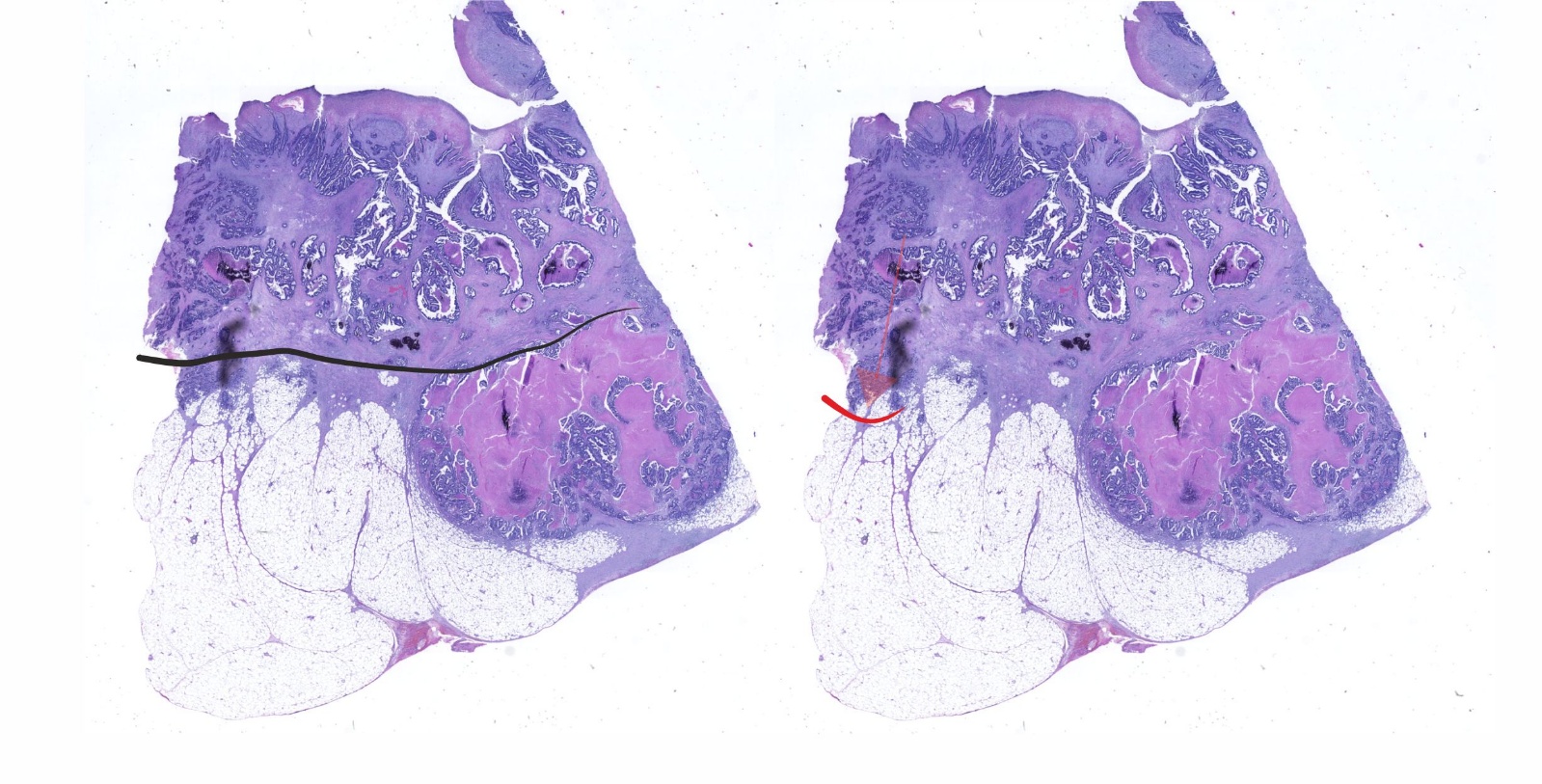


The black line represents the estimated line of the deepest anatomical layer that the cancer has completely passed, despite it being difficult to define in this case. The cancer penetrates the deepest layer in two different points, that are approximately as deep in their current layer. The chosen deepest point of invasion is the more infiltrative one and is marked with red.


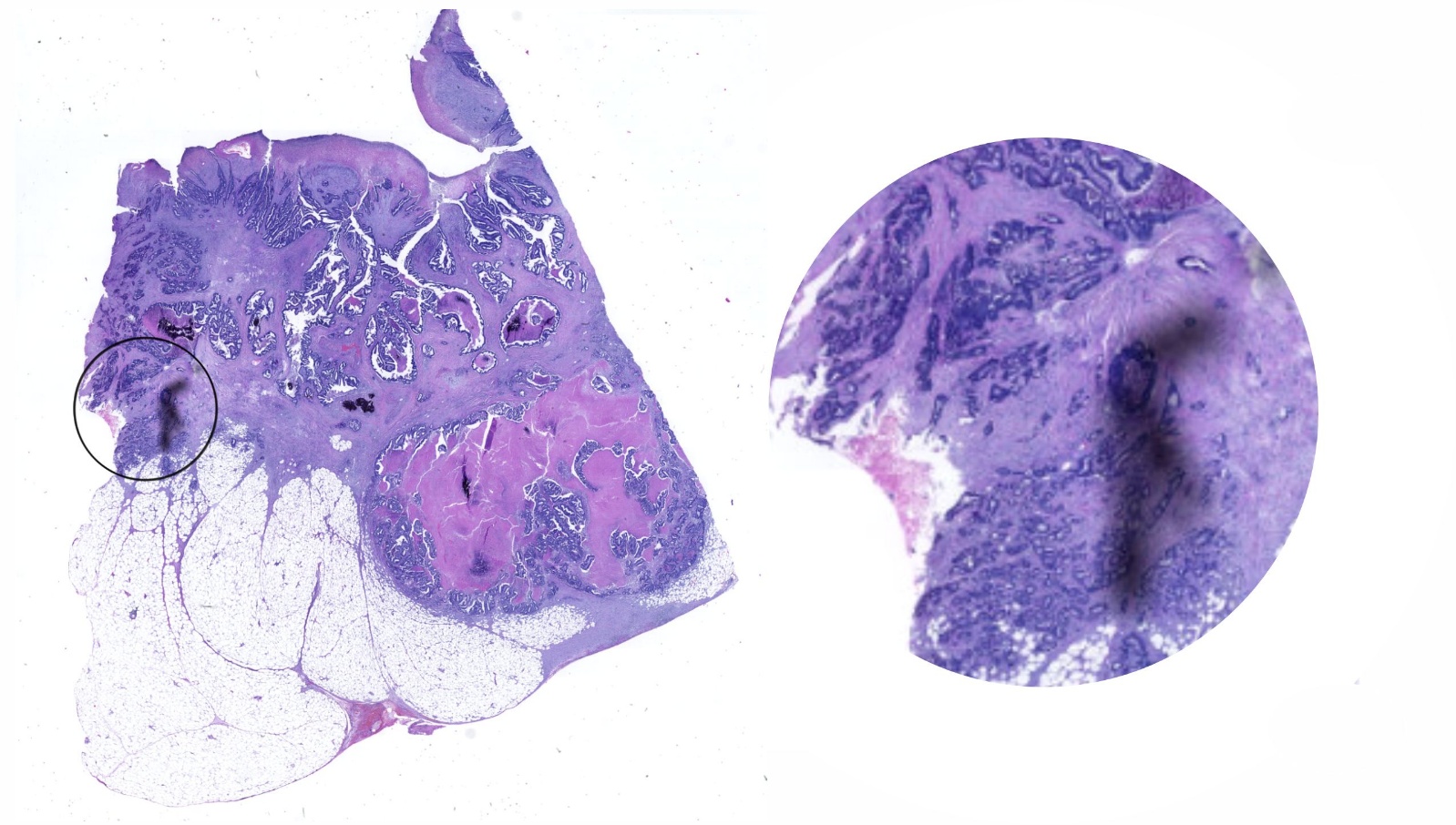


TIBI is estimated to be around 65%, placing it in the ‘intermediate’ category.

## Example 3


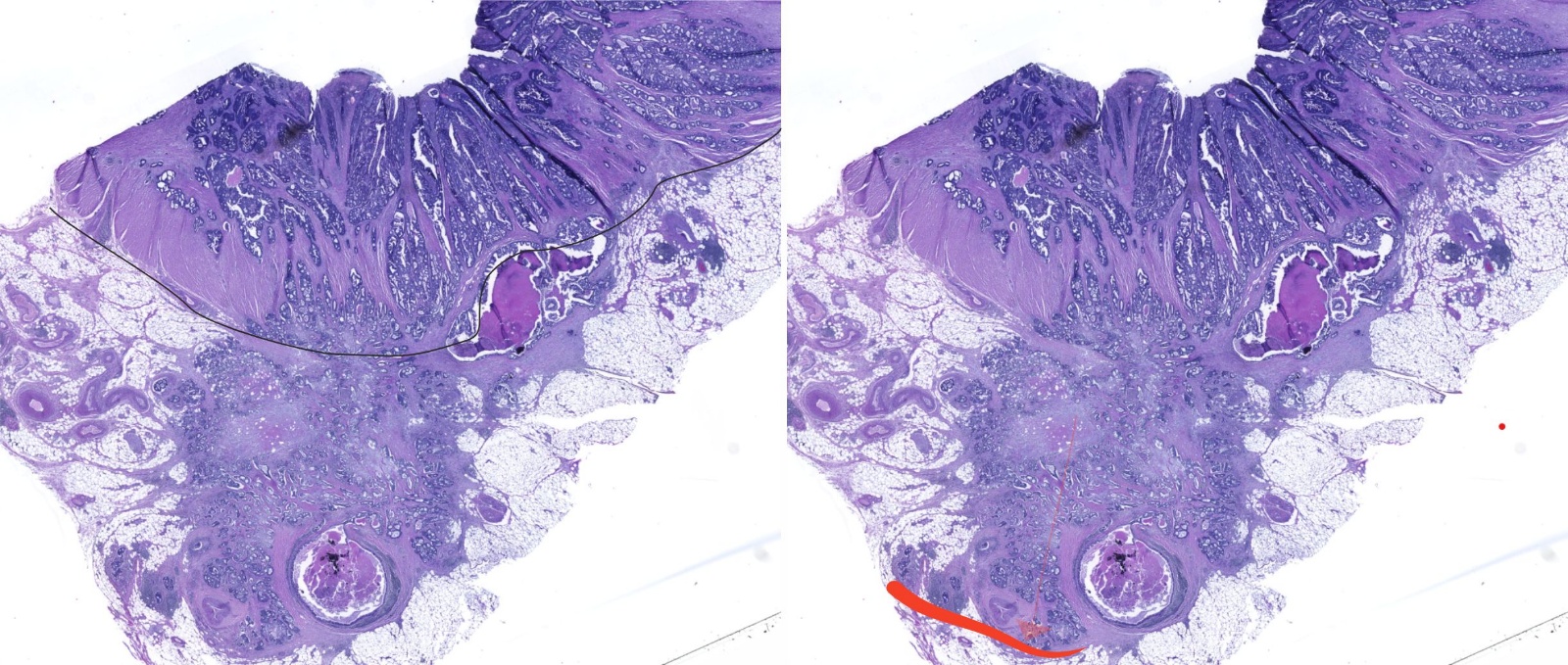


The black line represents the line of the deepest anatomical layer that the cancer has completely passed. The deepest point of invasion is marked with a red line.


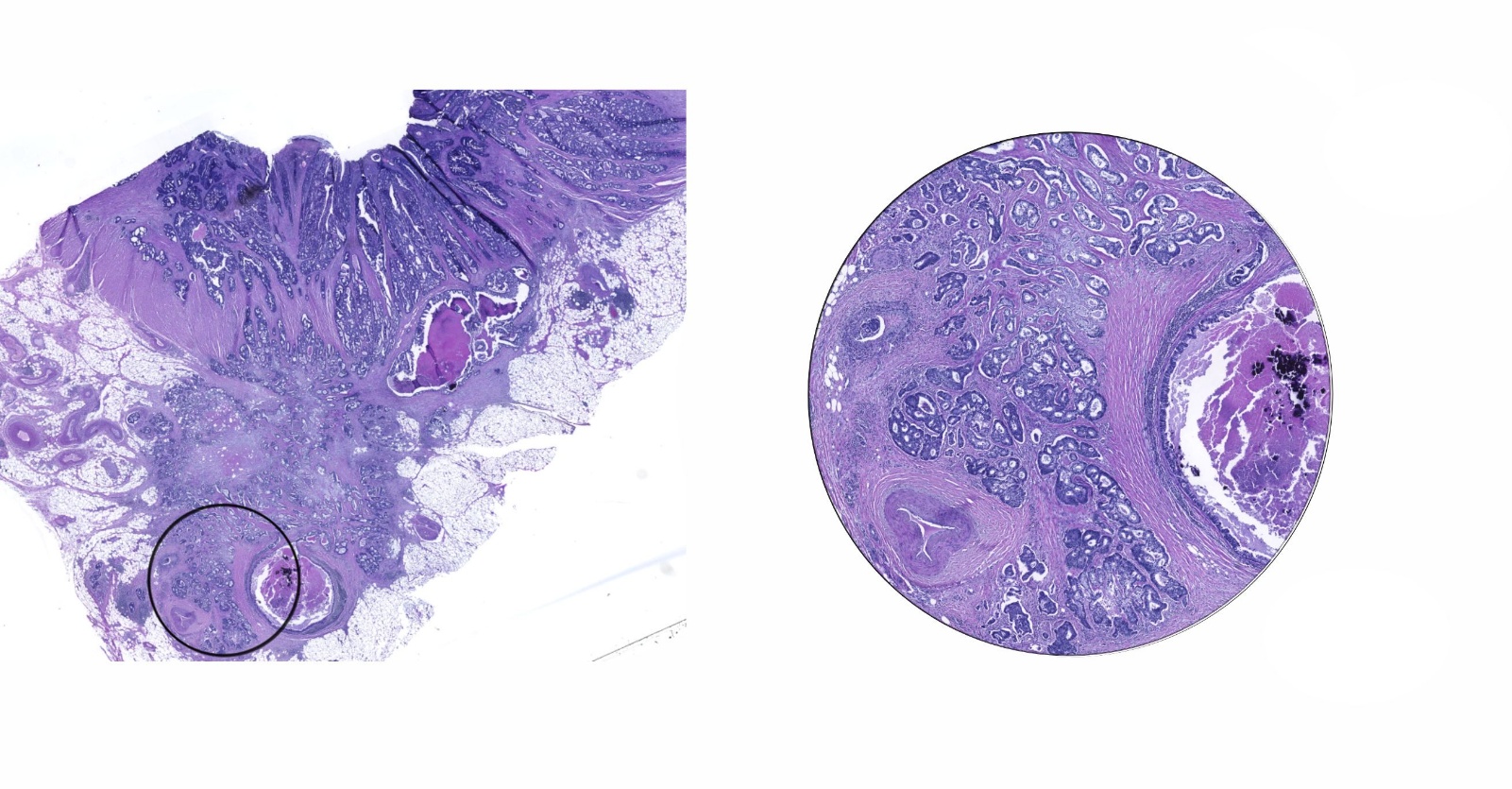


TIBI is estimated to be around 55%, placing it in the ‘intermediate’ category.

## Example 4


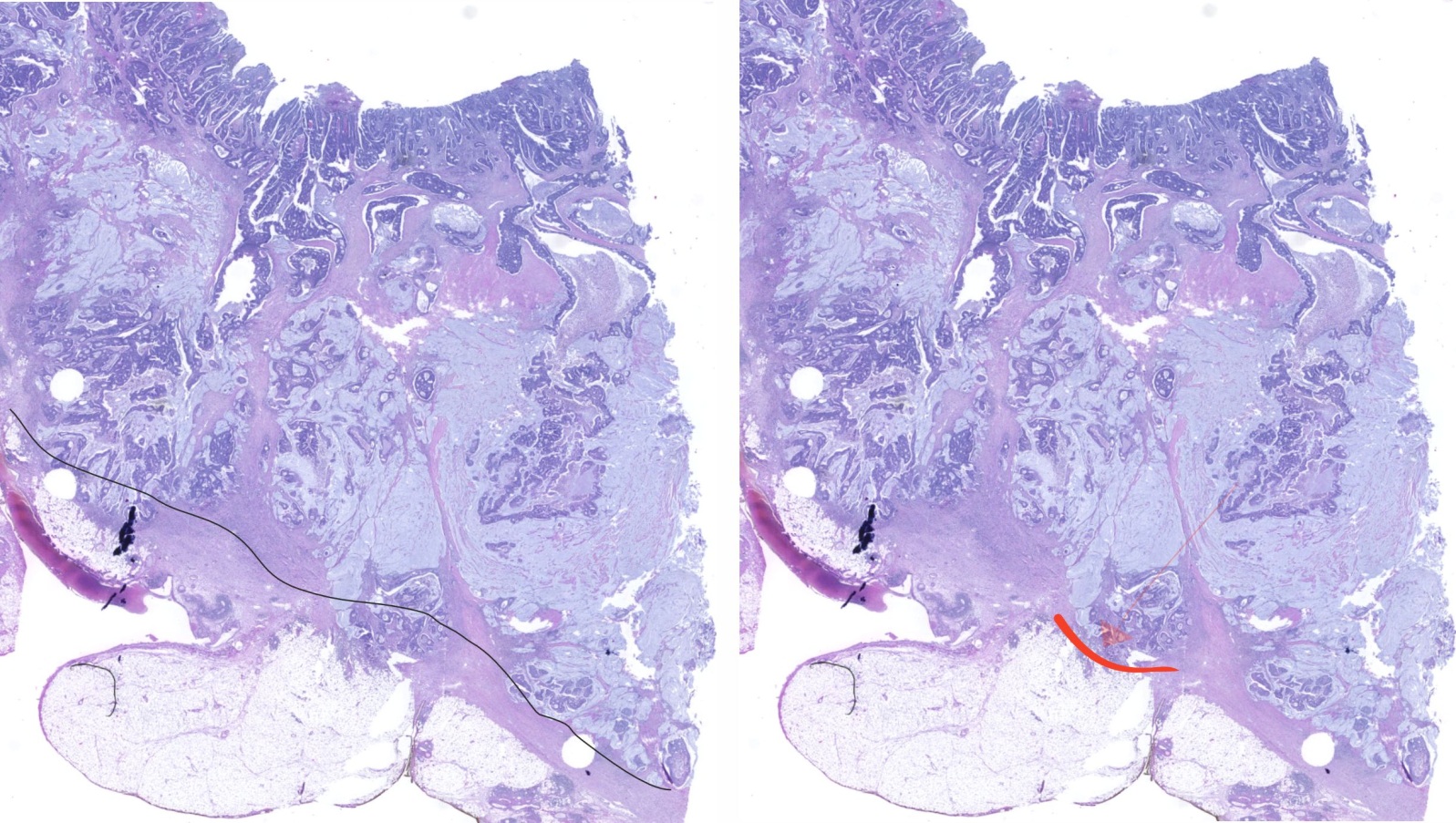


The black line represents the line of the deepest anatomical layer that the cancer has completely passed. The deepest point of invasion is marked with a red line.


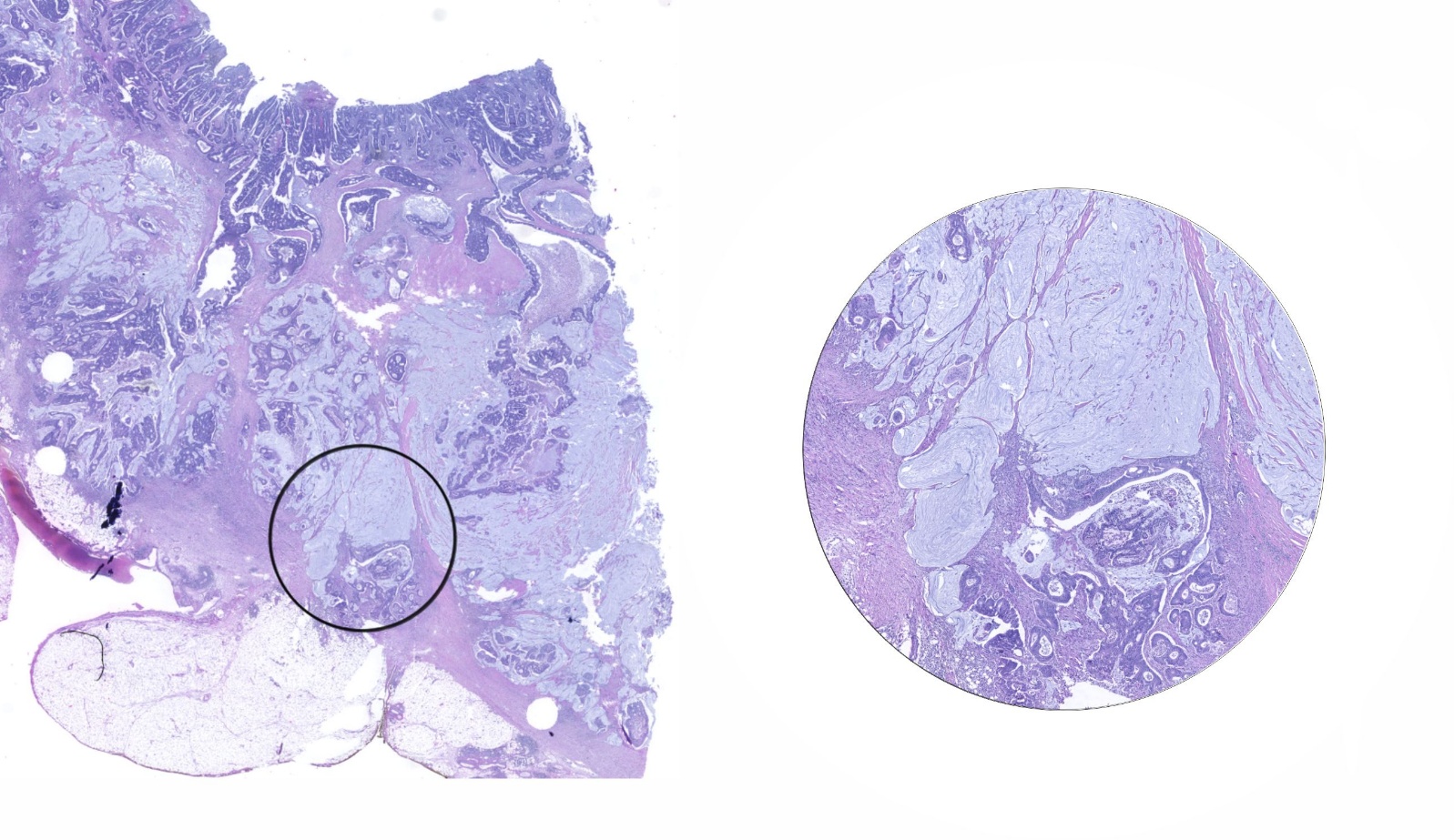


TIBI is estimated to be around 30%, placing it in the ‘low’ category.
